# Supplementary material for: Broad activation of latent HIV-1 in vivo
Source: Nat Commun. 2016 Sep 8;7:12731. doi: 10.1038/ncomms12731 (PMC5025526; doi:10.1038/ncomms12731)
Supplement: Supplementary Information — Supplementary Figures 1-31 and Supplementary Tables 1-4 [file ncomms12731-s1.pdf]

Supplementary figure 1

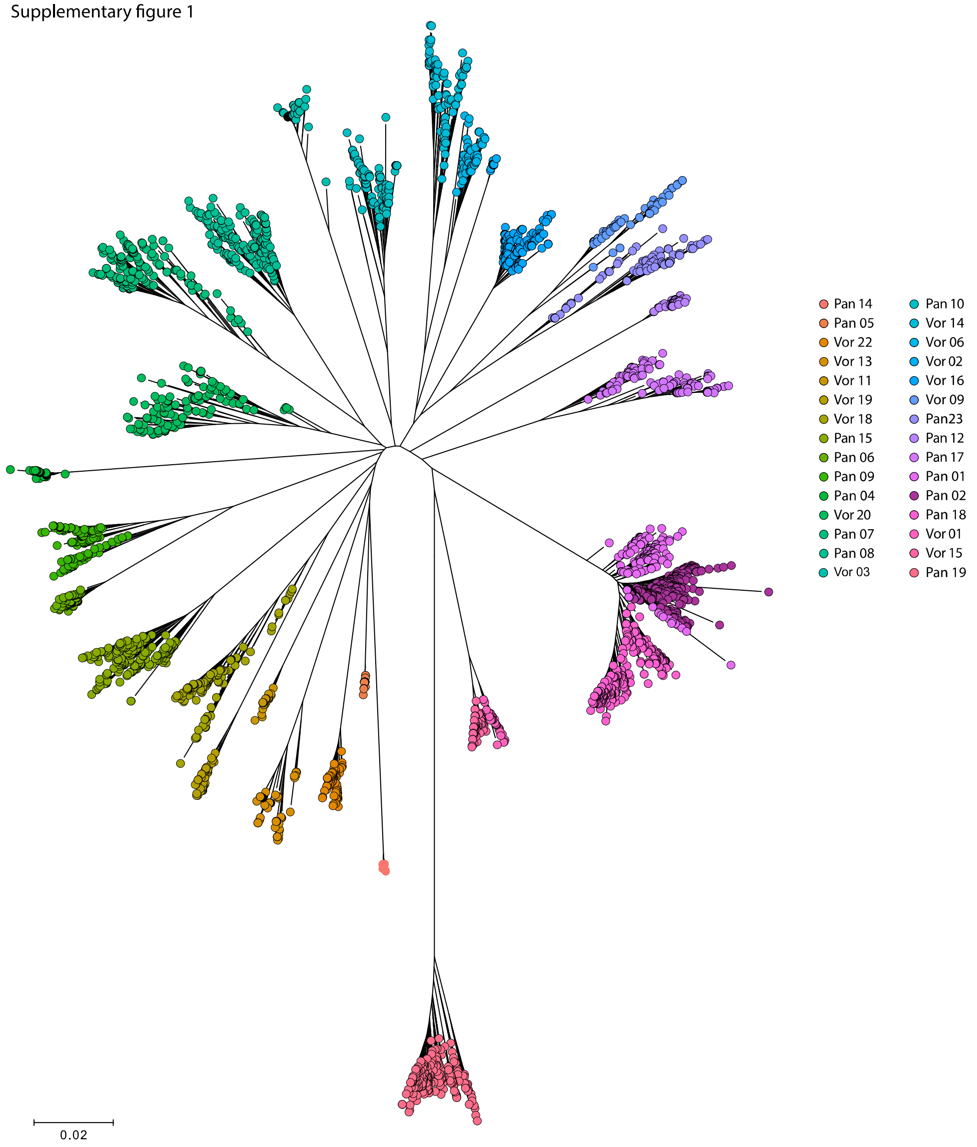

Supplementary Figure 1 Sequences from individual study participants segregate independently A neighbor-joining tree of sequences from all participants in both the panobinostat and vorinostat trials is shown. Sequences that were classified as defective (containing stop codons or hypermutation) were not included. The phylogenetic analysis and tree construction were conducted using MEGA-CC.

Supplementary figure 2

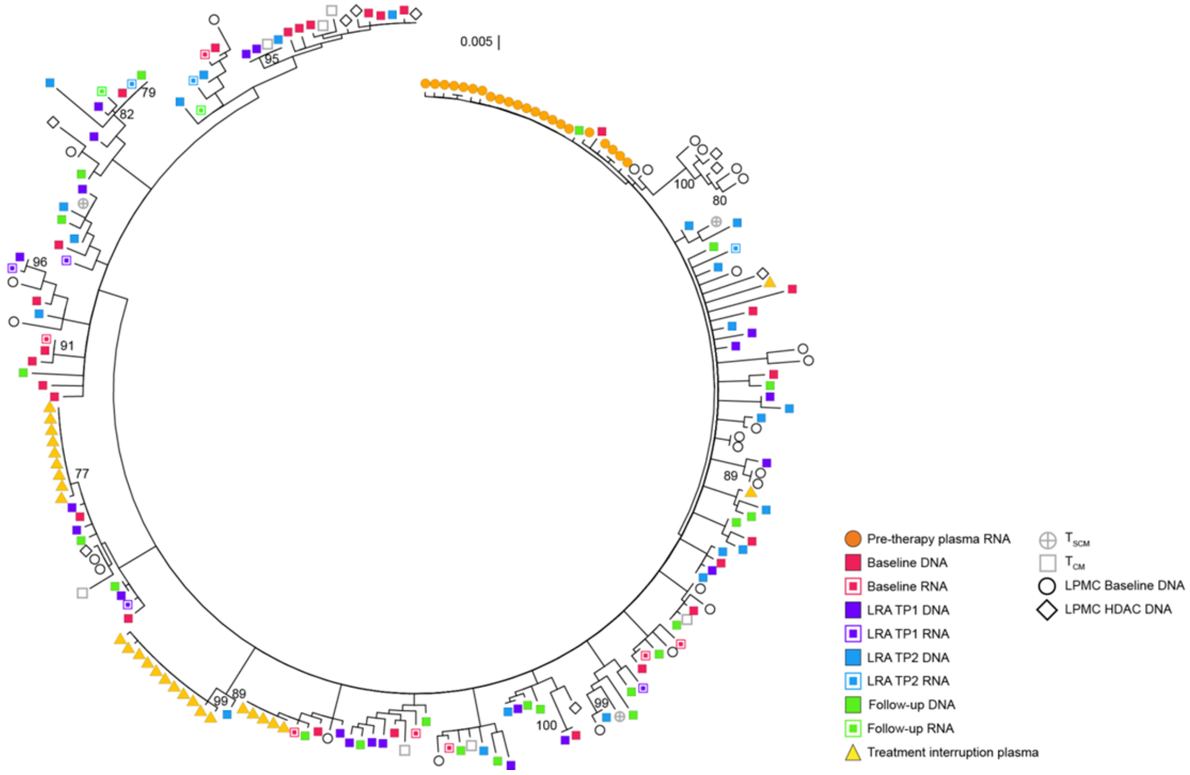

Supplementary Figure 2 Panobinostat participant 1 phylogenetic tree. Maximum-likelihood tree of the V1-V3 region of HIV-1 *env* sequences obtained using single-genome sequencing on isolated CD4+ T cells for participant 1 from the panobinostat trial. The phylogenetic analysis and tree construction were conducted using MEGA-CC. Defective sequences (hypermutants or containing stop codons) were excluded from the phylogenetic tree analysis. The plasma samples were collected approximately 2 months prior to initiation of anti-retroviral therapy and 14 days following the analytical treatment interruption. Peripheral blood samples were collected at baseline, 28 days after the first dose of panobinostat (TP1), 32 days after the first dose of panobinostat (TP2), and 38 days after the final panobinostat dose. Intestinal lamina propria mononuclear cells were collected at baseline (1 week prior to the first panobinostat dose) and during week 4 of the panobinostat trial. Cell subsets were sorted from peripheral blood samples collected 38 days after the final panobinostat dose.

Supplementary figure 3

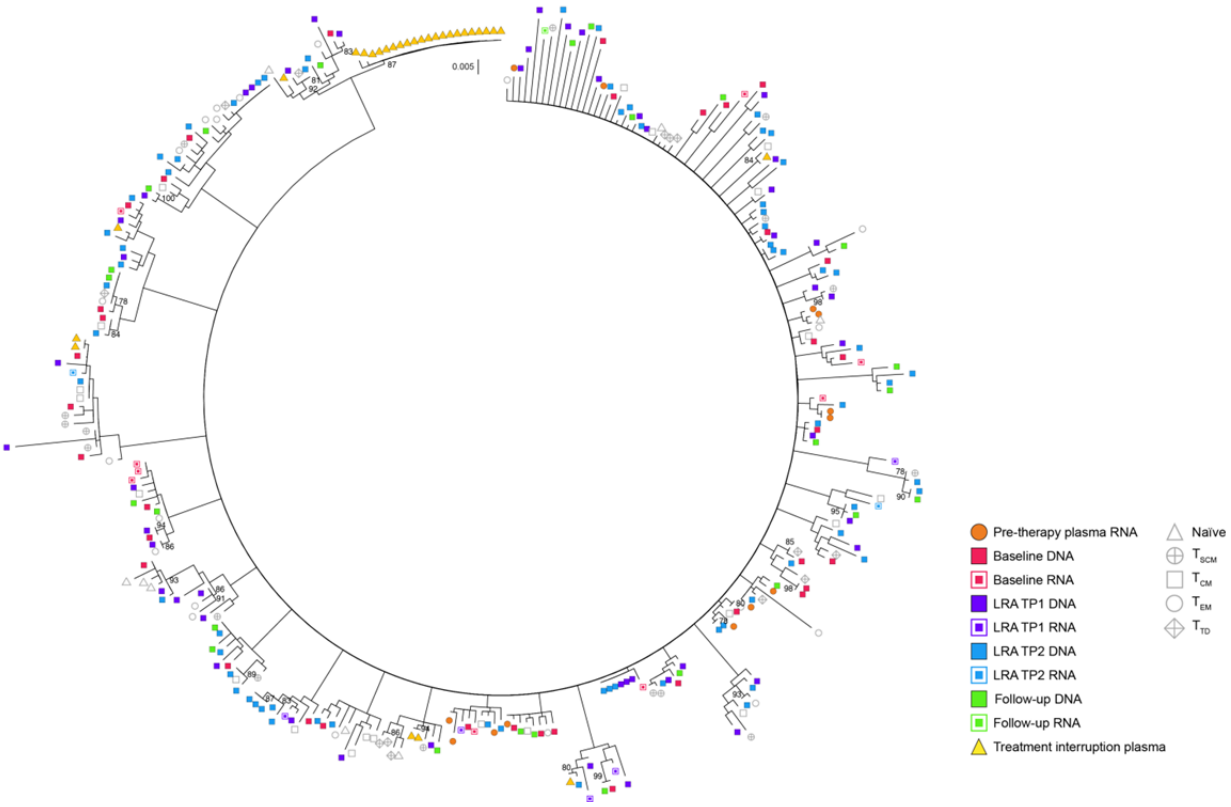

Supplementary Figure 3 Panobinostat participant 2 phylogenetic tree. Maximum-likelihood tree of the V1-V3 region of HIV-1 *env* sequences obtained using single-genome sequencing on isolated CD4+ T cells for participant 2 from the panobinostat trial. The phylogenetic analysis and tree construction were conducted using MEGA-CC. Defective sequences (hypermutants or containing stop codons) were excluded from the phylogenetic tree analysis. The plasma samples were collected approximately 1 year and 5 months prior to initiation of anti-retroviral therapy and 14 days following the analytical treatment interruption. Peripheral blood samples were collected at baseline, 2 hours after the first dose of panobinostat (TP1), 14 days and 42 days after the first dose of panobinostat (sequences from these two time points are both labeled as TP2), and 38 days after the final panobinostat dose. Cell subsets were sorted from peripheral blood samples collected 38 days after the final panobinostat dose.

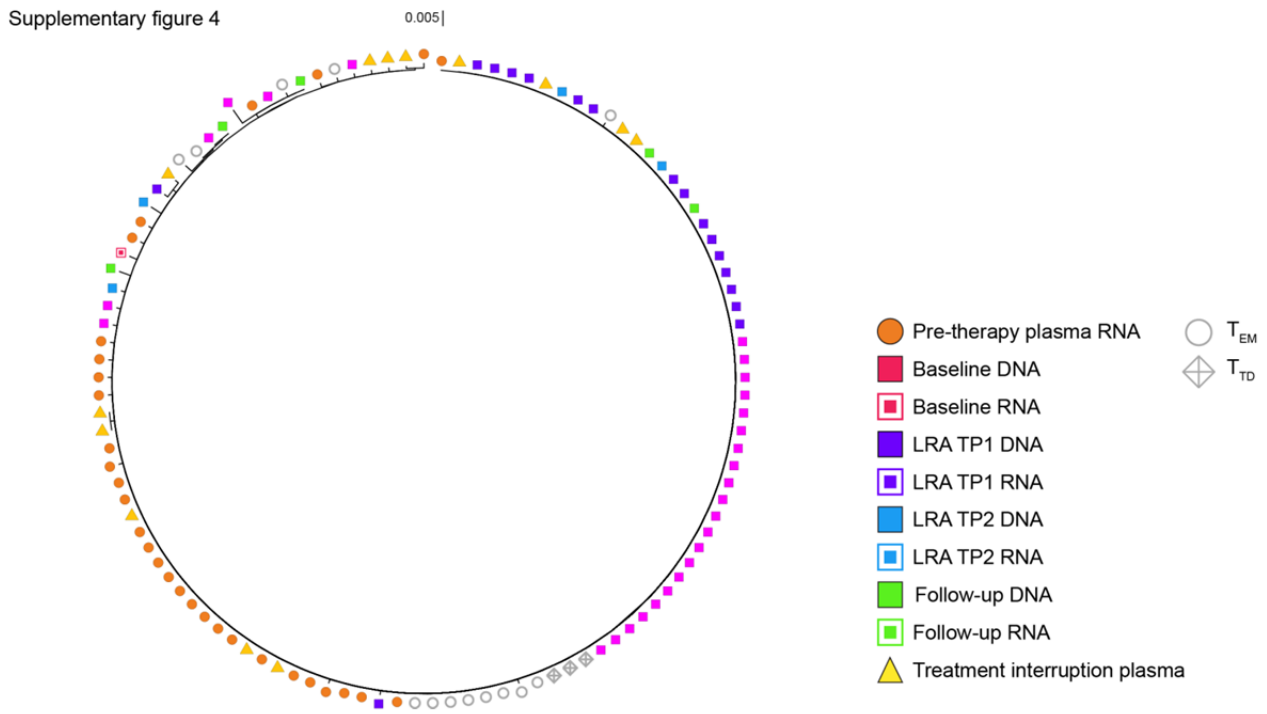

Supplementary Figure 4 Panobinostat participant 4 phylogenetic tree. Maximum-likelihood tree of the V1-V3 region of HIV-1 *env* sequences obtained using single-genome sequencing on isolated CD4+ T cells for participant 4 from the panobinostat trial. The phylogenetic analysis and tree construction were conducted using MEGA-CC. Defective sequences (hypermutants or containing stop codons) were excluded from the phylogenetic tree analysis. The plasma samples were collected the day of anti-retroviral therapy initiation and 46 and 55 days following the analytical treatment interruption. Peripheral blood samples were collected at baseline, 1 day after the first dose of panobinostat (TP1), 28 days after the first dose of panobinostat (TP2), and 38 days after the final panobinostat dose. Cell subsets were sorted from peripheral blood samples collected 38 days after the final panobinostat dose.

Supplementary figure 5

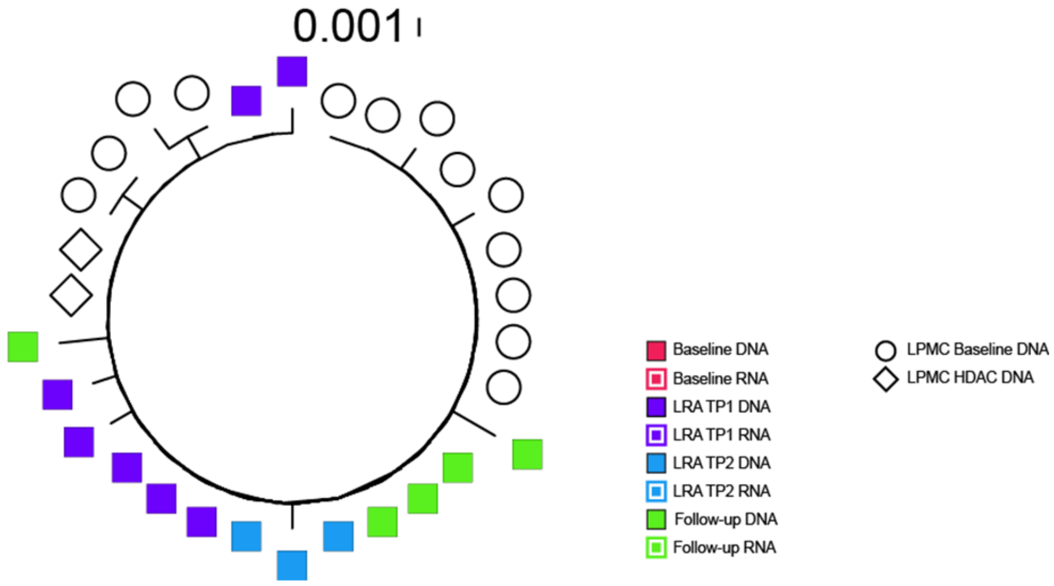

Supplementary Figure 5 Panobinostat participant 5 phylogenetic tree. Maximum-likelihood tree of the V1-V3 region of HIV-1 *env* sequences obtained using single-genome sequencing on isolated CD4+ T cells for participant 5 from the panobinostat trial. The phylogenetic analysis and tree construction were conducted using MEGA-CC. Defective sequences (hypermutants or containing stop codons) were excluded from the phylogenetic tree analysis. Peripheral blood samples were collected at baseline, 2 hours after the first dose of panobinostat (TP1), 28 days after the first dose of panobinostat (TP2), and 38 days after the final panobinostat dose. Intestinal lamina propria mononuclear cells were collected at baseline (1 week prior to the first panobinostat dose) and during week 4 of the panobinostat trial.

Supplementary figure 6

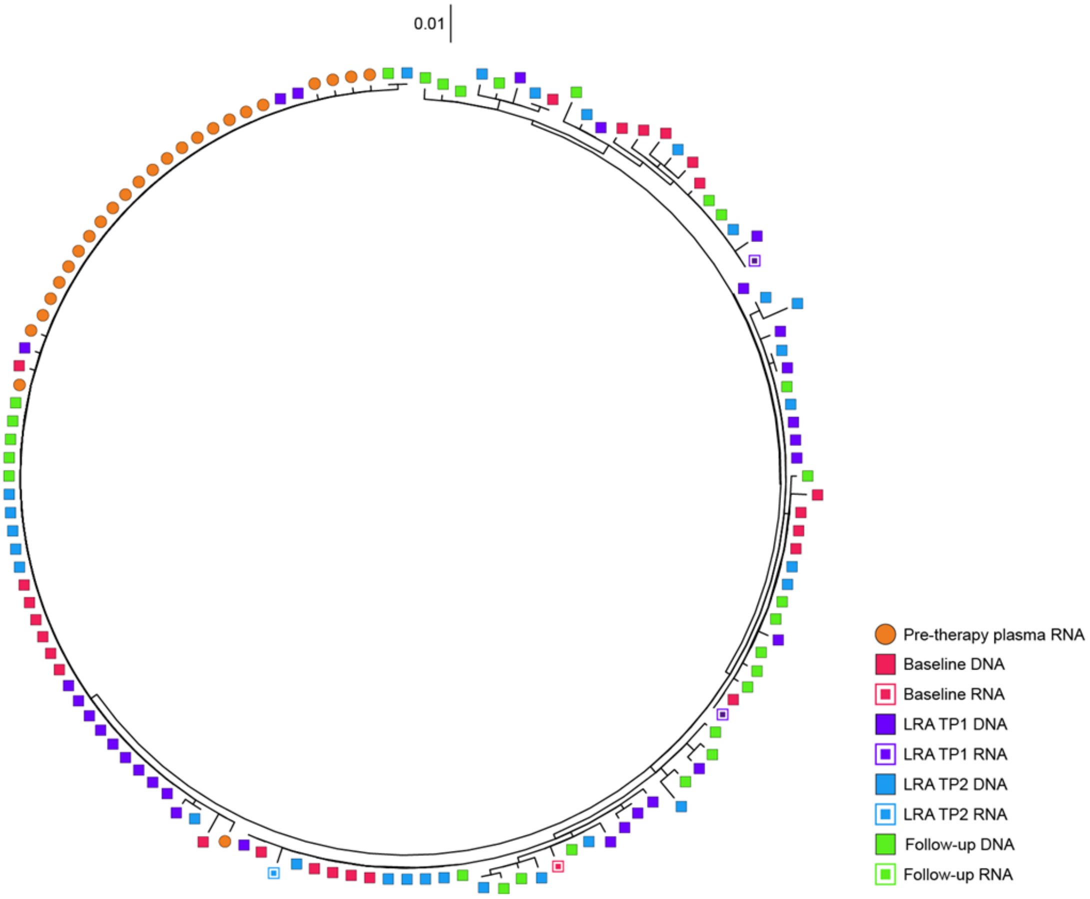

Supplementary Figure 6 Panobinostat participant 6 phylogenetic tree. Maximum-likelihood tree of the V1-V3 region of HIV-1 *env* sequences obtained using single-genome sequencing on isolated CD4+ T cells for participant 6 from the panobinostat trial. The phylogenetic analysis and tree construction were conducted using MEGA-CC. Defective sequences (hypermutants or containing stop codons) were excluded from the phylogenetic tree analysis. The plasma sample was collected approximately 8 months prior to initiation of anti-retroviral therapy. Peripheral blood samples were collected at baseline, 2 hours after the first dose of panobinostat (TP1), 1 day after the first dose of panobinostat (TP2), and 38 days after the final panobinostat dose.

Supplementary figure 7

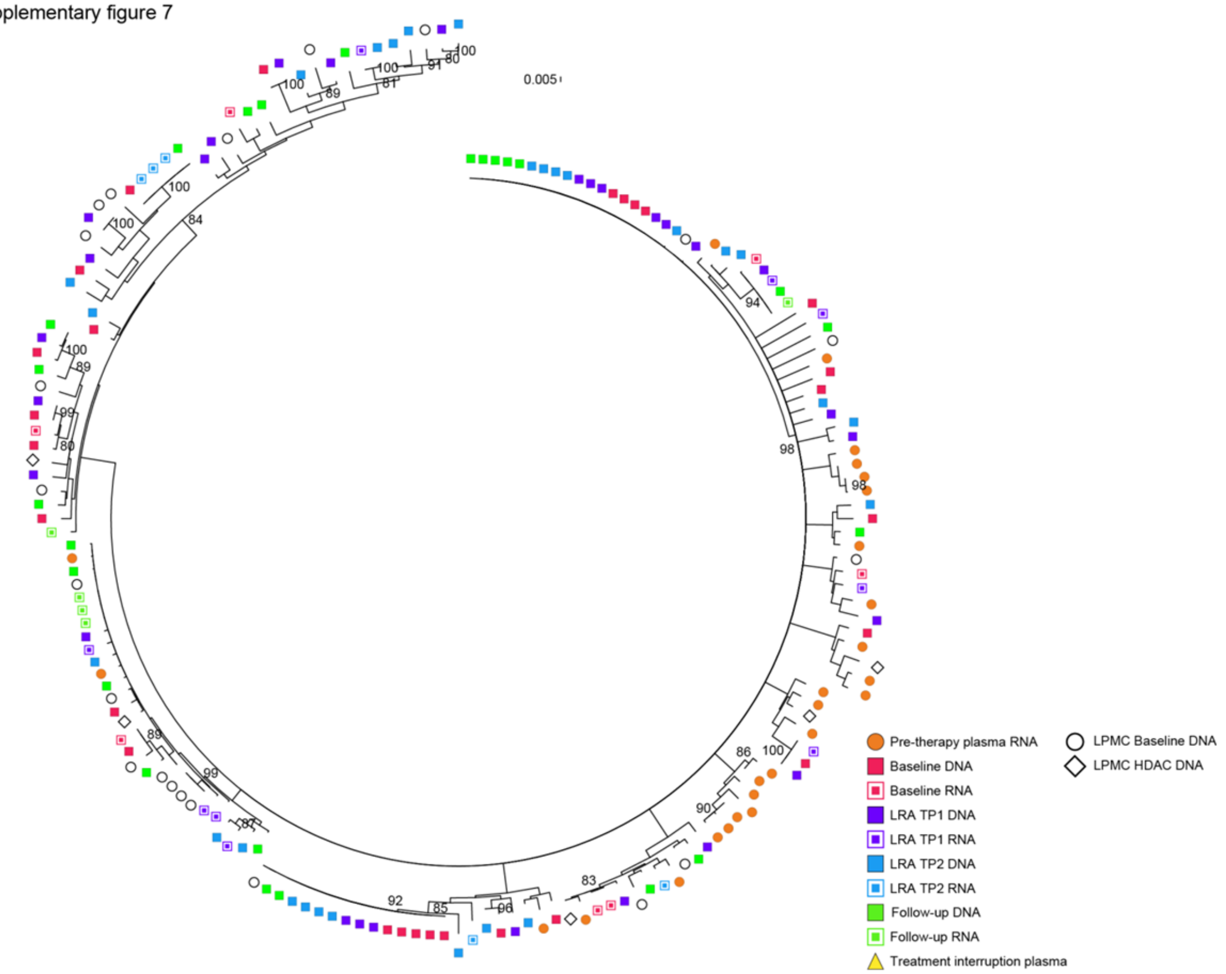

Supplementary Figure 7 Panobinostat participant 7 phylogenetic tree. Maximum-likelihood tree of the V1-V3 region of HIV-1 *env* sequences obtained using single-genome sequencing on isolated CD4+ T cells for participant 7 from the panobinostat trial. The phylogenetic analysis and tree construction were conducted using MEGA-CC. Defective sequences (hypermutants or containing stop codons) were excluded from the phylogenetic tree analysis. The plasma sample was collected approximately 1 year prior to initiation of anti-retroviral therapy. Peripheral blood samples were collected at baseline, 2 hours after the first dose of panobinostat (TP1), 28 days after the first dose of panobinostat (TP2), and 38 days after the final panobinostat dose. Intestinal lamina propria mononuclear cells were collected at baseline (1 week prior to the first panobinostat dose) and during week 4 of the panobinostat trial.

Supplementary figure 8

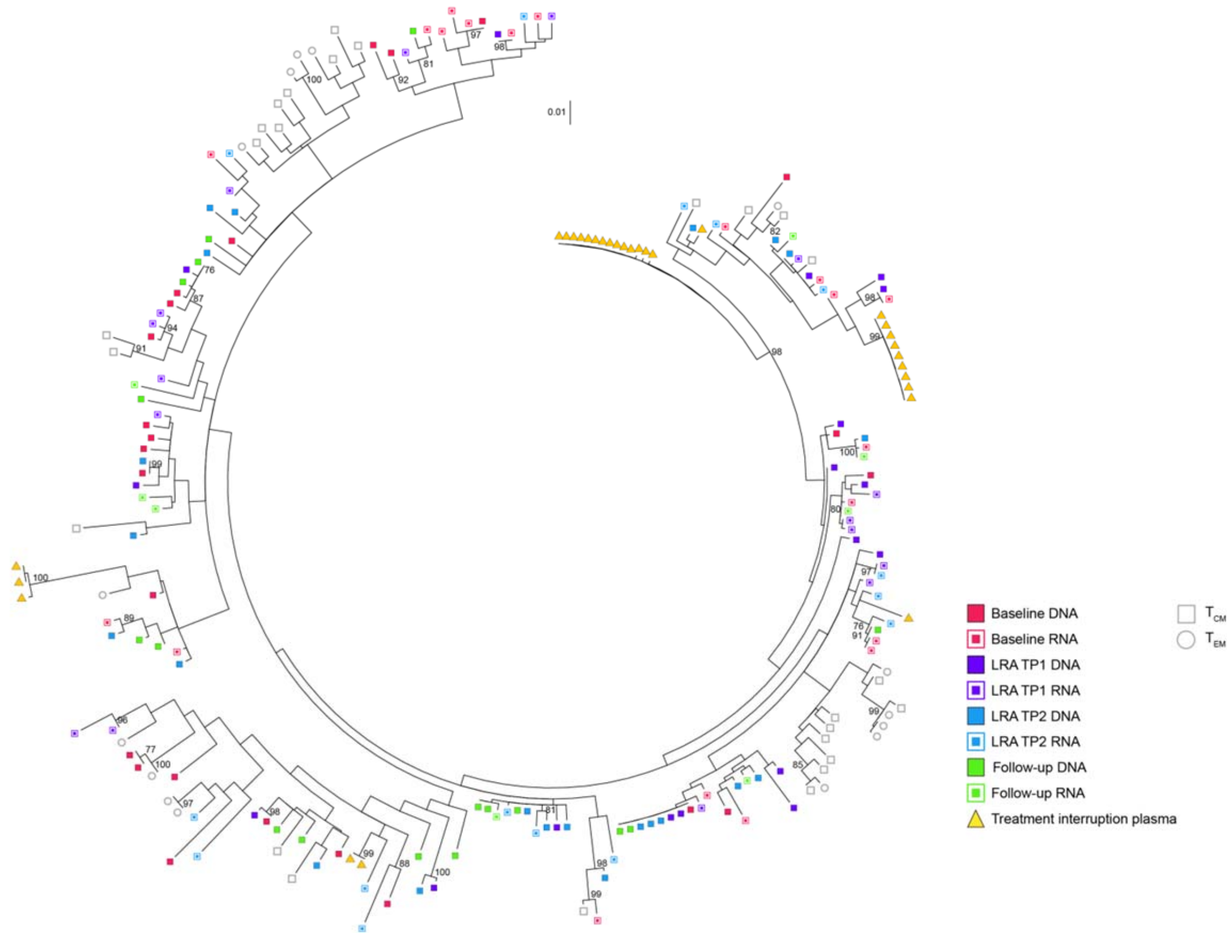

Supplementary Figure 8 Panobinostat participant 8 phylogenetic tree. Maximum-likelihood tree of the V1-V3 region of HIV-1 *env* sequences obtained using single-genome sequencing on isolated CD4+ T cells for participant 8 from the panobinostat trial. The phylogenetic analysis and tree construction were conducted using MEGA-CC. Defective sequences (hypermutants or containing stop codons) were excluded from the phylogenetic tree analysis. The plasma sample was collected 14 days following the analytical treatment interruption. Peripheral blood samples were collected at baseline, 2 hours after the first dose of panobinostat (TP1), 32 days after the first dose of panobinostat (TP2), and 38 days after the final panobinostat dose. Cell subsets were sorted from peripheral blood samples collected 38 days after the final panobinostat dose.

Supplementary figure 9

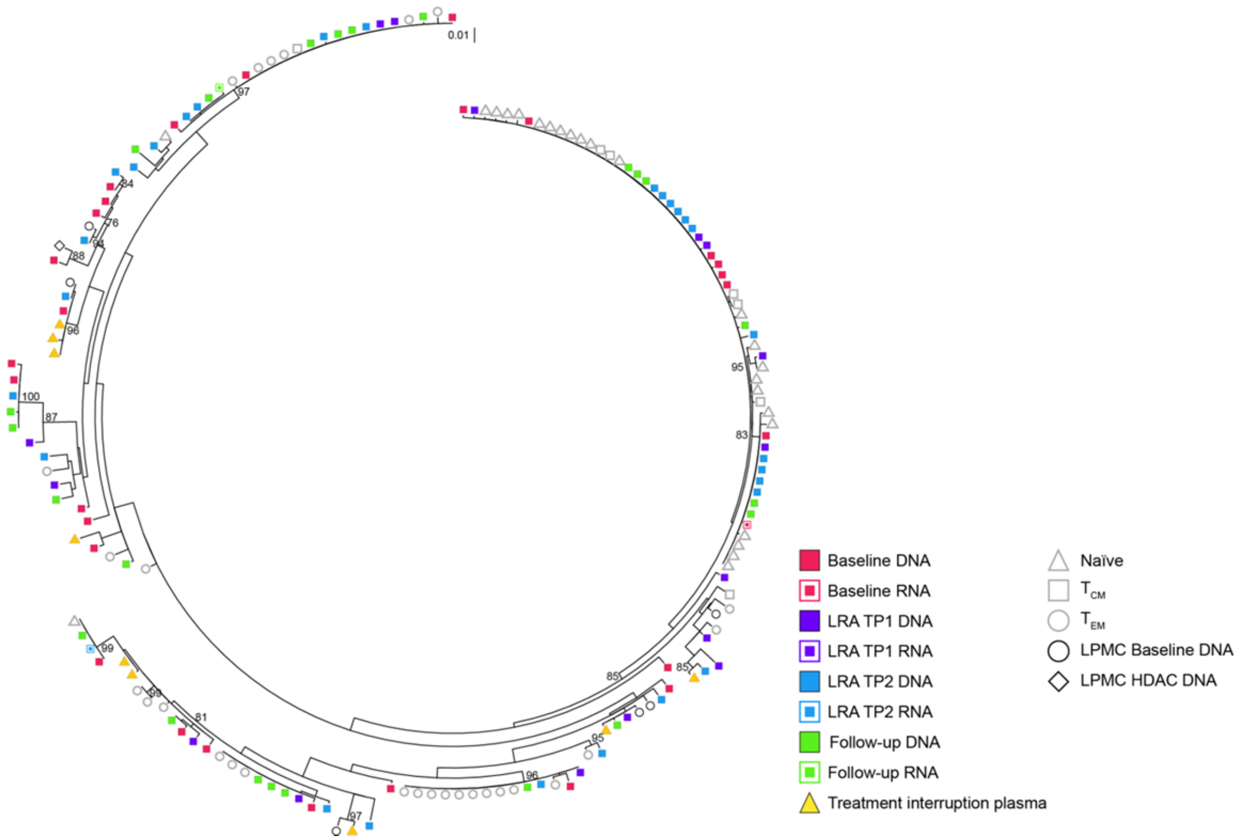

Supplementary Figure 9 Panobinostat participant 9 phylogenetic tree. Maximum-likelihood tree of the V1-V3 region of HIV-1 *env* sequences obtained using single-genome sequencing on isolated CD4+ T cells for participant 9 from the panobinostat trial. The phylogenetic analysis and tree construction were conducted using MEGA-CC. Defective sequences (hypermutants or containing stop codons) were excluded from the phylogenetic tree analysis. The plasma sample was collected 24 days following the analytical treatment interruption. Peripheral blood samples were collected at baseline, 14 days after the first dose of panobinostat (TP1), 28 days after the first dose of panobinostat (TP2), and 38 days after the final panobinostat dose. Intestinal lamina propria mononuclear cells were collected at baseline (1 week prior to the first panobinostat dose) and during week 4 of the panobinostat trial. Cell subsets were sorted from peripheral blood samples collected 38 days after the final panobinostat dose.

Supplementary figure 10

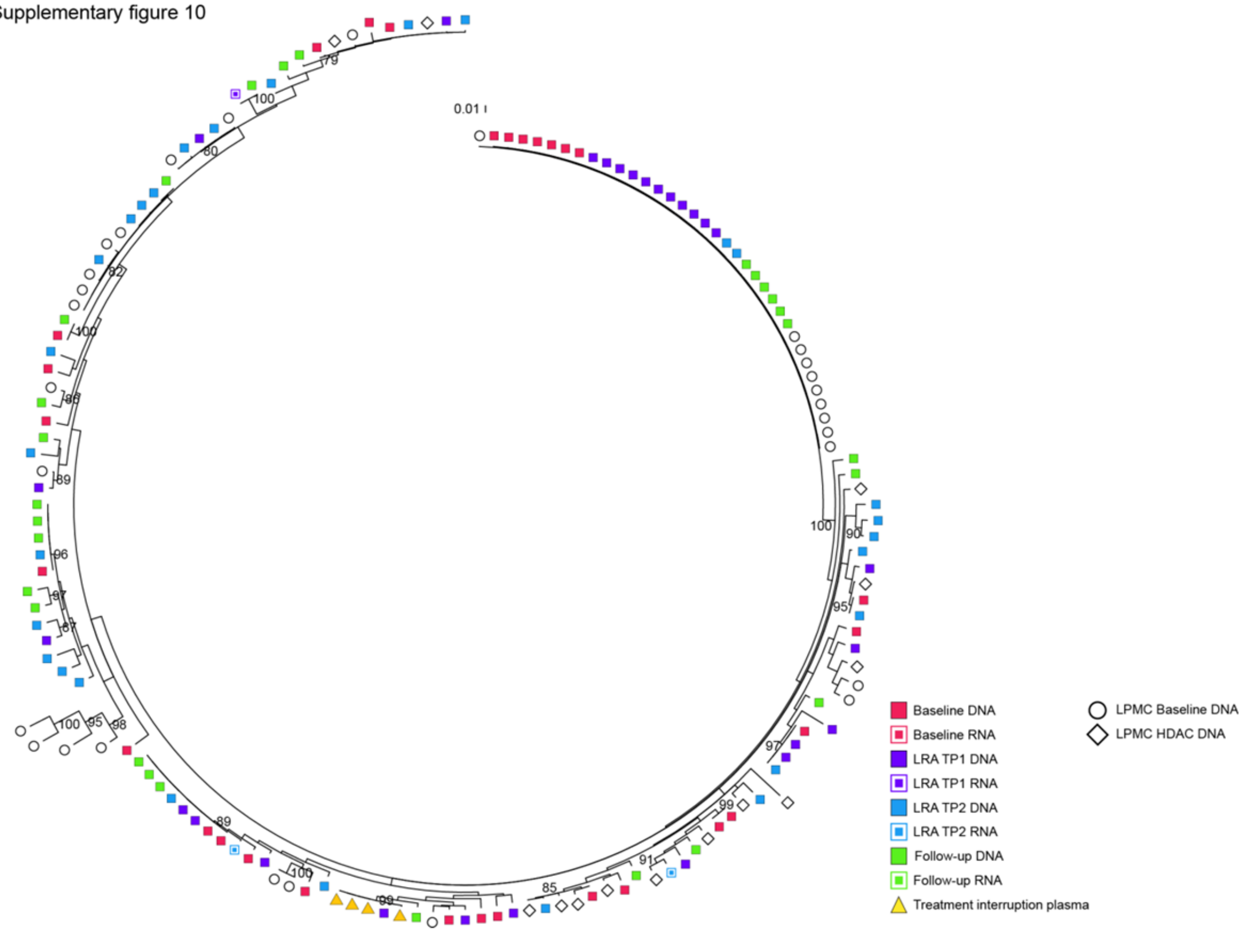

Supplementary Figure 10 Panobinostat participant 10 phylogenetic tree. Maximum-likelihood tree of the V1-V3 region of HIV-1 *env* sequences obtained using single-genome sequencing on isolated CD4+ T cells for participant 10 from the panobinostat trial. The phylogenetic analysis and tree construction were conducted using MEGA-CC. Defective sequences (hypermutants or containing stop codons) were excluded from the phylogenetic tree analysis. The plasma sample was collected 145 days following the analytical treatment interruption. Peripheral blood samples were collected at baseline, 1 day after the first dose of panobinostat (TP1), 28 days after the first dose of panobinostat (TP2), and 38 days after the final panobinostat dose. Intestinal lamina propria mononuclear cells were collected at baseline (1 week prior to the first panobinostat dose) and during week 4 of the panobinostat trial.

Supplementary figure 11

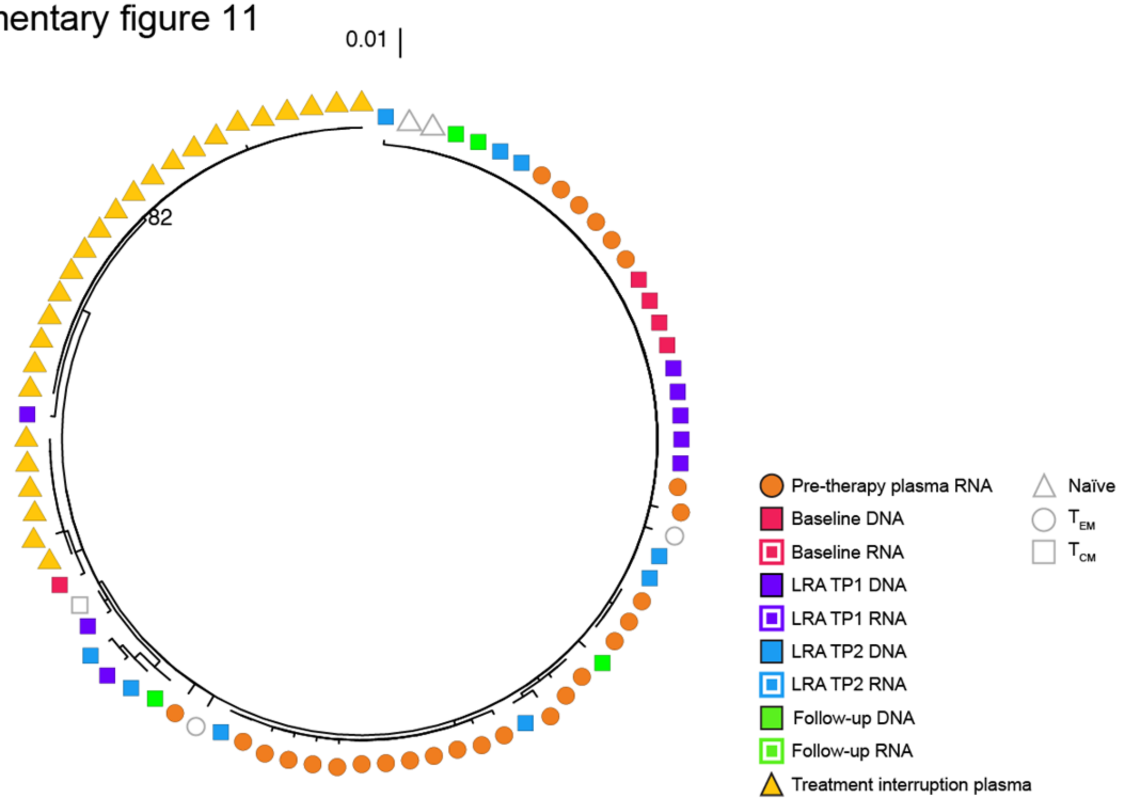

Supplementary Figure 11 Panobinostat participant 12 phylogenetic tree. Maximum-likelihood tree of the V1-V3 region of HIV-1 *env* sequences obtained using single-genome sequencing on isolated CD4+ T cells for participant 12 from the panobinostat trial. The phylogenetic analysis and tree construction were conducted using MEGA-CC. Defective sequences (hypermutants or containing stop codons) were excluded from the phylogenetic tree analysis. The plasma samples were collected approximately 1 year and 2 months prior to initiation of anti-retroviral therapy and 17 days following the analytical treatment interruption. Peripheral blood samples were collected at baseline, 28 days after the first dose of panobinostat (TP1), 46 days after the first dose of panobinostat (TP2), and 38 days after the final panobinostat dose. Cell subsets were sorted from peripheral blood samples collected 38 days after the final panobinostat dose.

Supplementary figure 12

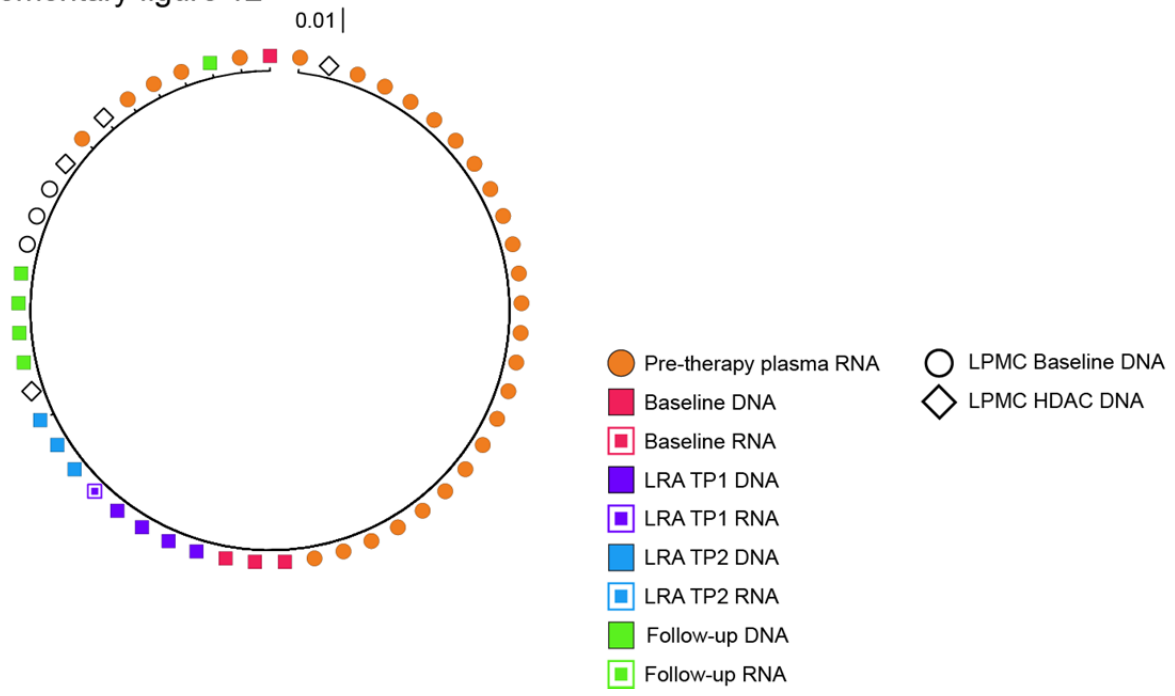

Supplementary Figure 12 Panobinostat participant 14 phylogenetic tree. Maximum-likelihood tree of the V1-V3 region of HIV-1 *env* sequences obtained using single-genome sequencing on isolated CD4+ T cells for participant 14 from the panobinostat trial. The phylogenetic analysis and tree construction were conducted using MEGA-CC. Defective sequences (hypermutants or containing stop codons) were excluded from the phylogenetic tree analysis. The plasma samples was collected 2 days prior to initiation of anti-retroviral therapy. Peripheral blood samples were collected at baseline, 9 days after the first dose of panobinostat (TP1), 18 days after the first dose of panobinostat (TP2), and 38 days after the final panobinostat dose. Intestinal lamina propria mononuclear cells were collected at baseline (1 week prior to the first panobinostat dose) and during week 4 of the panobinostat trial.

Supplementary figure 13

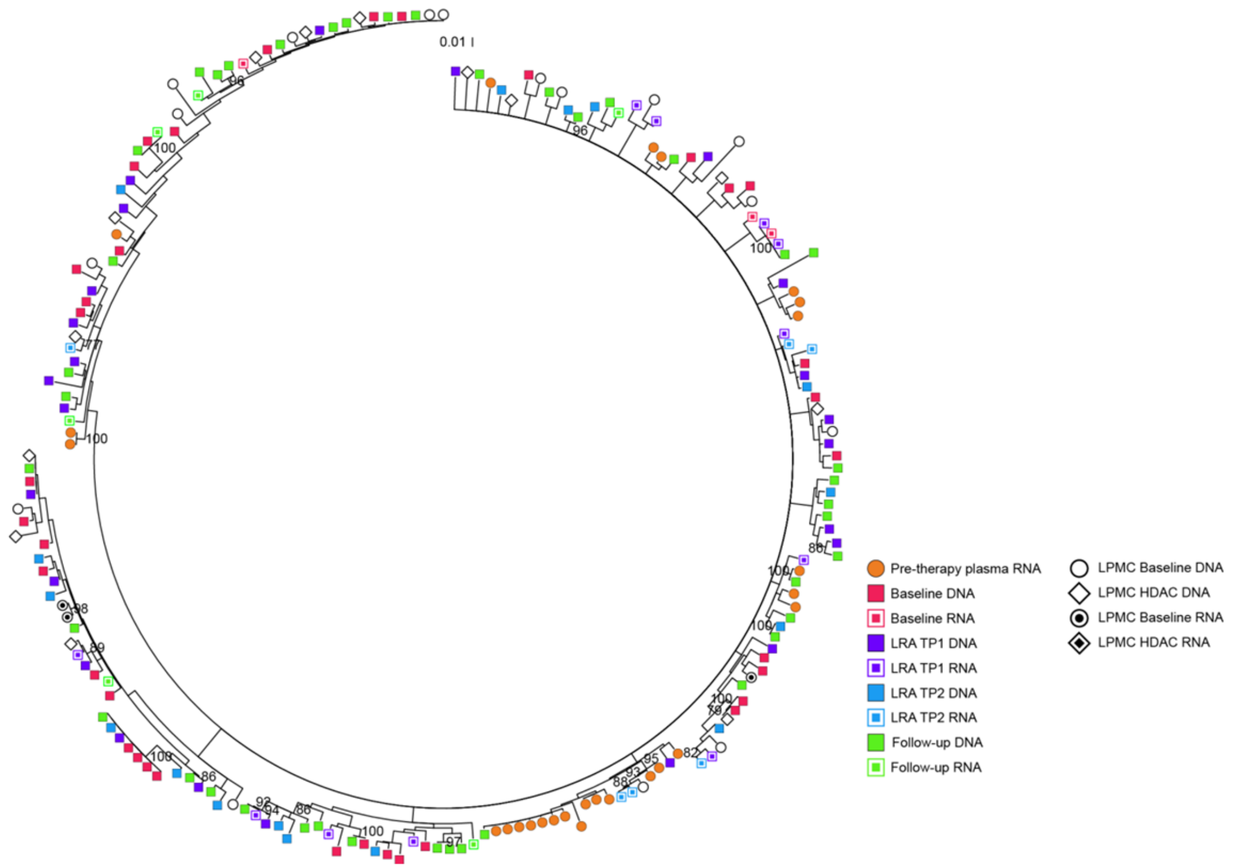

Supplementary Figure 13 Panobinostat participant 15 phylogenetic tree. Maximum-likelihood tree of the V1-V3 region of HIV-1 *env* sequences obtained using single-genome sequencing on isolated CD4+ T cells for participant 15 from the panobinostat trial. The phylogenetic analysis and tree construction were conducted using MEGA-CC. Defective sequences (hypermutants or containing stop codons) were excluded from the phylogenetic tree analysis. The plasma sample was collected approximately 2 years and 1 month prior to initiation of anti-retroviral therapy. Peripheral blood samples were collected at baseline, 32 days after the first dose of panobinostat (TP1), 46 days after the first dose of panobinostat (TP2), and 38 days after the final panobinostat dose. Intestinal lamina propria mononuclear cells were collected at baseline (1 week prior to the first panobinostat dose) and during week 4 of the panobinostat trial.

Supplementary figure 14

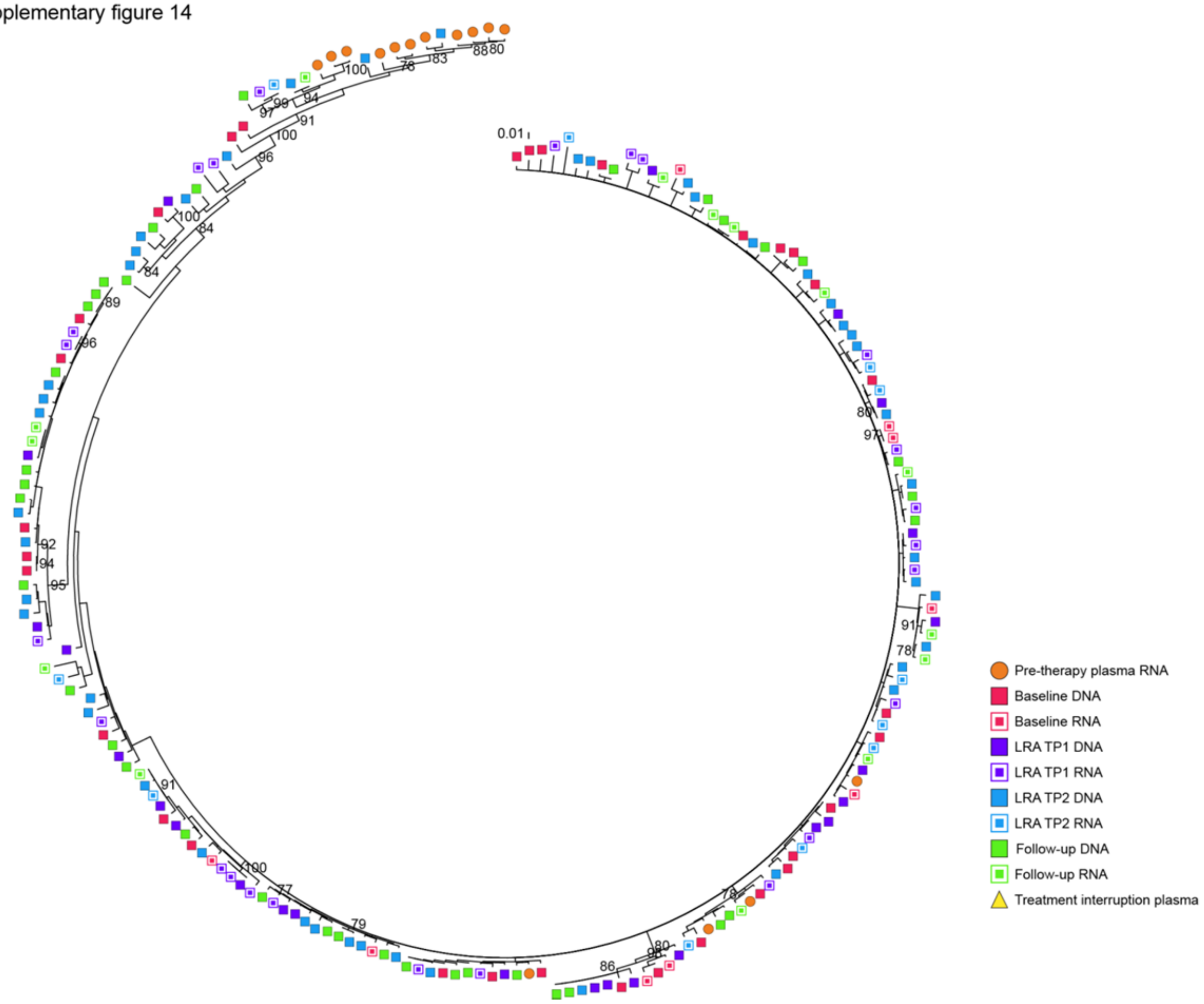

Supplementary Figure 14 Panobinostat participant 19 phylogenetic tree. Maximum-likelihood tree of the V1-V3 region of HIV-1 *env* sequences obtained using single-genome sequencing on isolated CD4+ T cells for participant 19 from the panobinostat trial. The phylogenetic analysis and tree construction were conducted using MEGA-CC. Defective sequences (hypermutants or containing stop codons) were excluded from the phylogenetic tree analysis. The plasma sample was collected approximately 9 months prior to initiation of anti-retroviral therapy. Peripheral blood samples were collected at baseline, 23 days after the first dose of panobinostat (TP1), 32 days after the first dose of panobinostat (TP2), and 38 days after the final panobinostat dose.

Supplementary figure 15

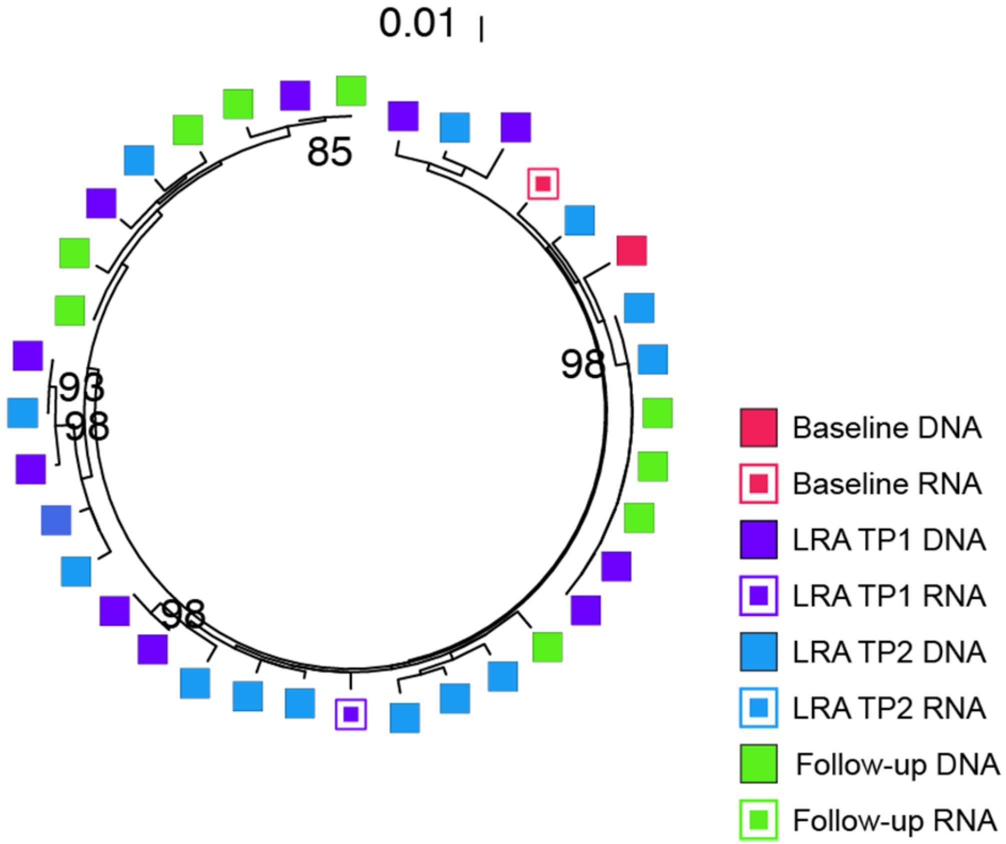

Supplementary Figure 15 Vorinostat participant VOR001 phylogenetic tree. Maximum-likelihood tree of the V1-V3 region of HIV-1 *env* sequences obtained using single-genome sequencing on isolated CD4+ T cells for participant VOR001 from the vorinostat trial. The phylogenetic analysis and tree construction were conducted using MEGA-CC. Defective sequences (hypermutants or containing stop codons) were excluded from the phylogenetic tree analysis. Peripheral blood samples were collected at baseline, 8 hours after the first dose of vorinostat (TP1), 14 days after the first dose of vorinostat (TP2), and 7 days after the final vorinostat dose.

Supplementary figure 16

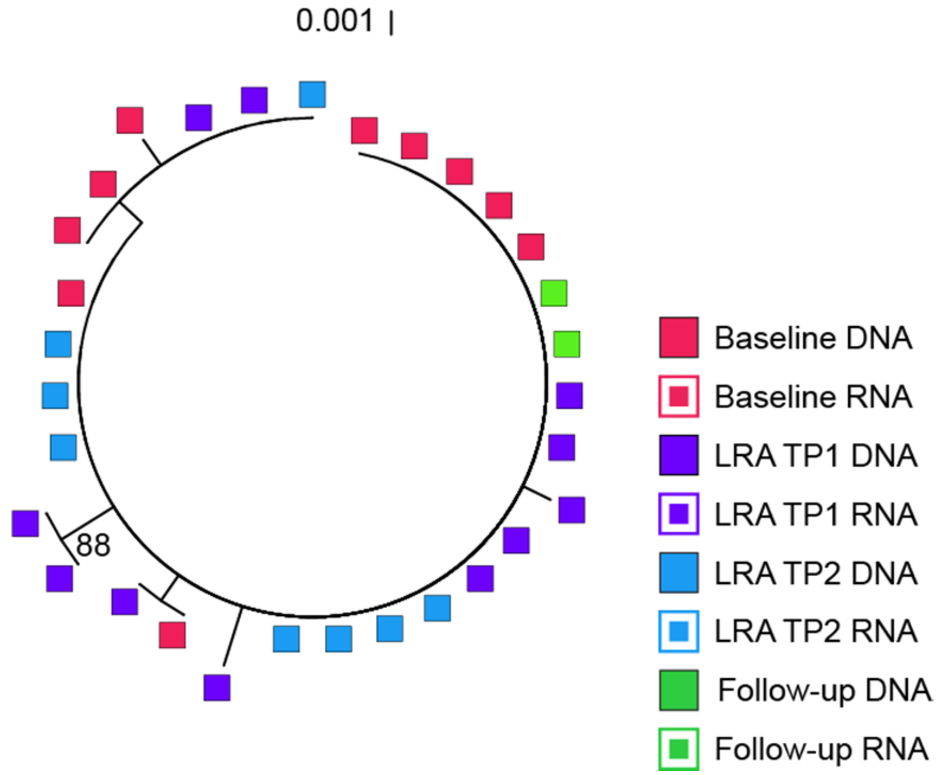

Supplementary Figure 16 Vorinostat participant VOR002 phylogenetic tree. Maximum-likelihood tree of the V1-V3 region of HIV-1 *env* sequences obtained using single-genome sequencing on isolated CD4+ T cells for participant VOR002 from the vorinostat trial. The phylogenetic analysis and tree construction were conducted using MEGA-CC. Defective sequences (hypermutants or containing stop codons) were excluded from the phylogenetic tree analysis. Peripheral blood samples were collected at baseline, 24 hours after the first dose of vorinostat (TP1), 7 days after the first dose of vorinostat (TP2), and 7 days after the final vorinostat dose.

Supplementary figure 17

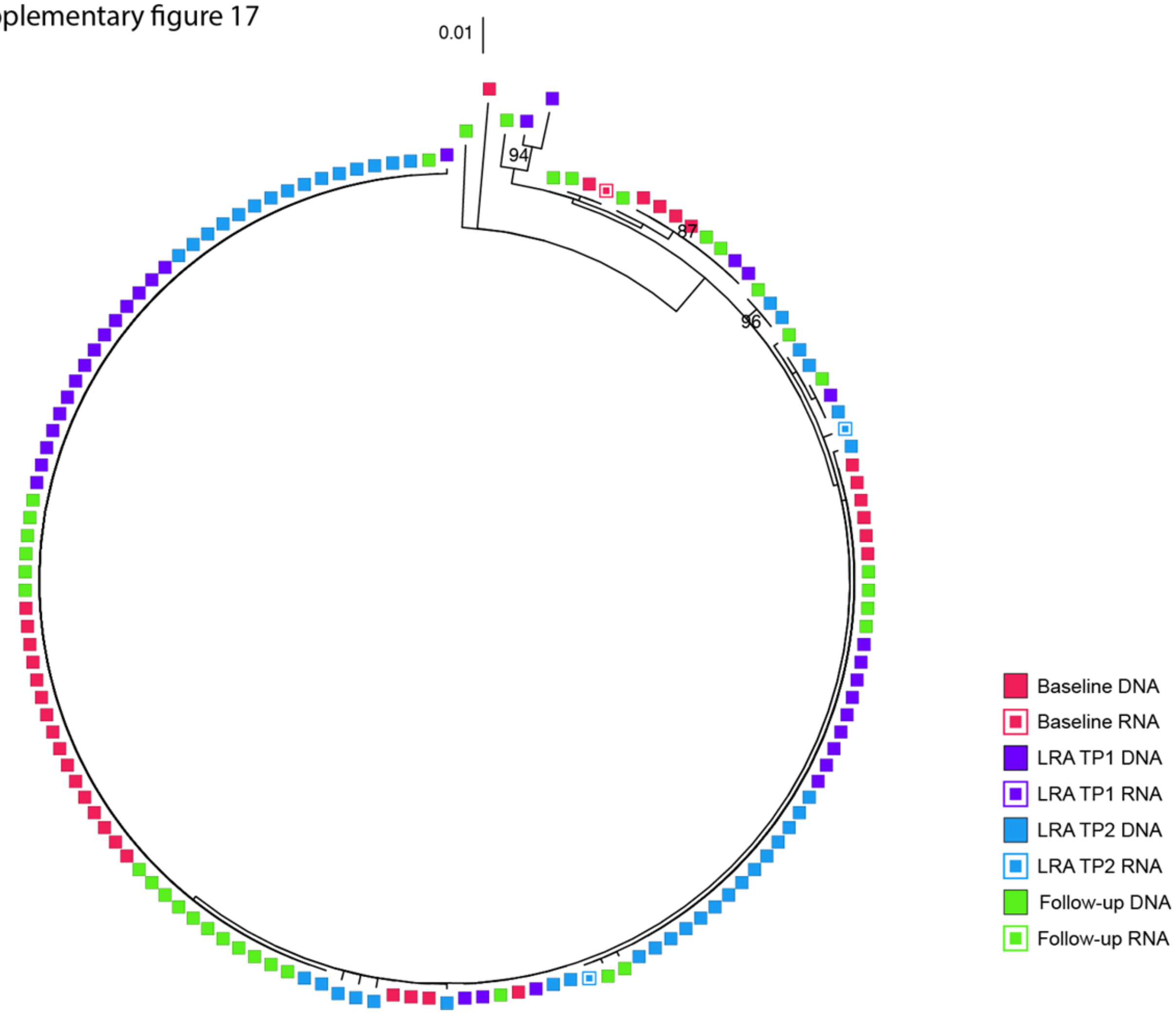

Supplementary Figure 17 Vorinostat participant VOR003 phylogenetic tree. Maximum-likelihood tree of the V1-V3 region of HIV-1 *env* sequences obtained using single-genome sequencing on isolated CD4+ T cells for participant VOR003 from the vorinostat trial. The phylogenetic analysis and tree construction were conducted using MEGA-CC. Defective sequences (hypermutants or containing stop codons) were excluded from the phylogenetic tree analysis. Peripheral blood samples were collected at baseline, 2 hours after the first dose of vorinostat (TP1), 24 hours after the first dose of vorinostat (TP2), and 7 days after the final vorinostat dose.

Supplementary figure 18

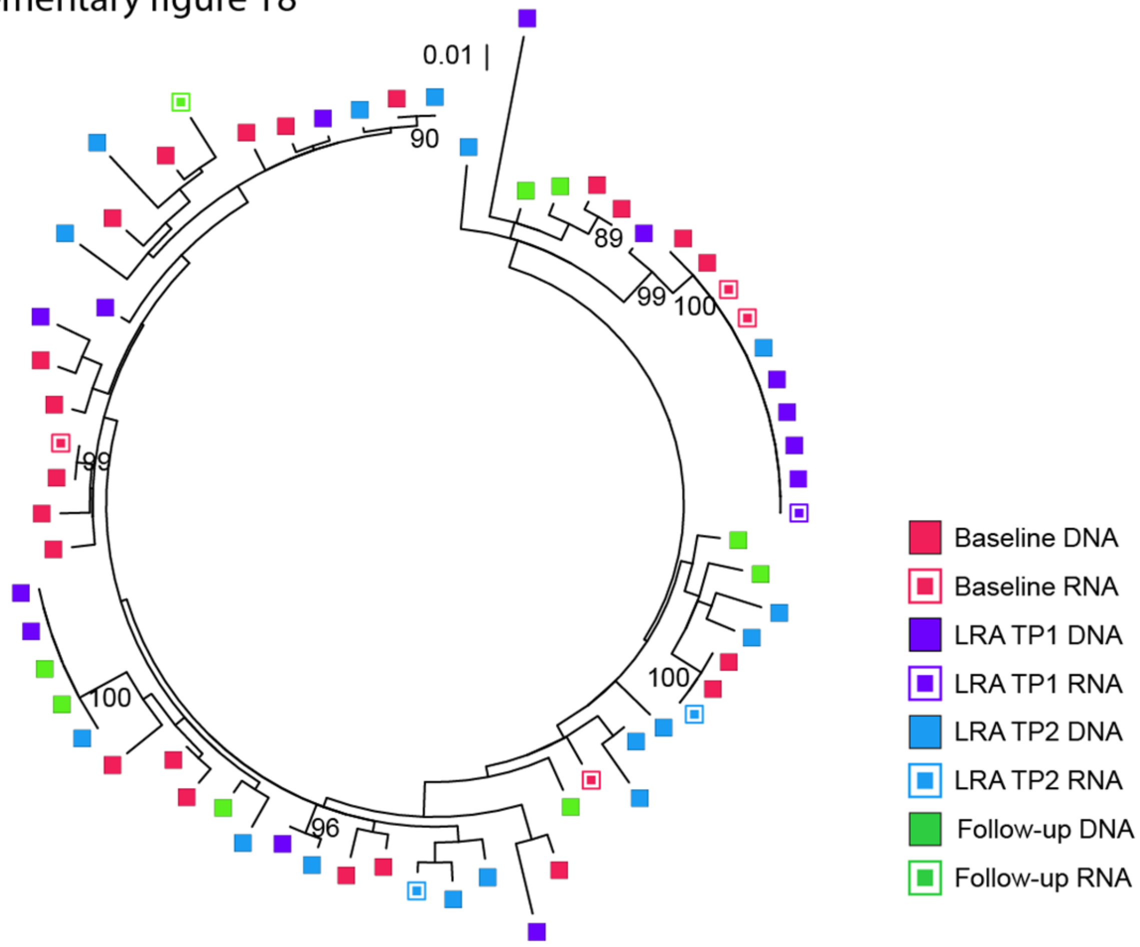

Supplementary Figure 18 Vorinostat participant VOR006 phylogenetic tree. Maximum-likelihood tree of the V1-V3 region of HIV-1 *env* sequences obtained using single-genome sequencing on isolated CD4+ T cells for participant VOR006 from the vorinostat trial. The phylogenetic analysis and tree construction were conducted using MEGA-CC. Defective sequences (hypermutants or containing stop codons) were excluded from the phylogenetic tree analysis. Peripheral blood samples were collected at baseline, 7 days after the first dose of vorinostat (TP1), 14 days after the first dose of vorinostat (TP2), and 7 days after the final vorinostat dose.

Supplementary figure 19

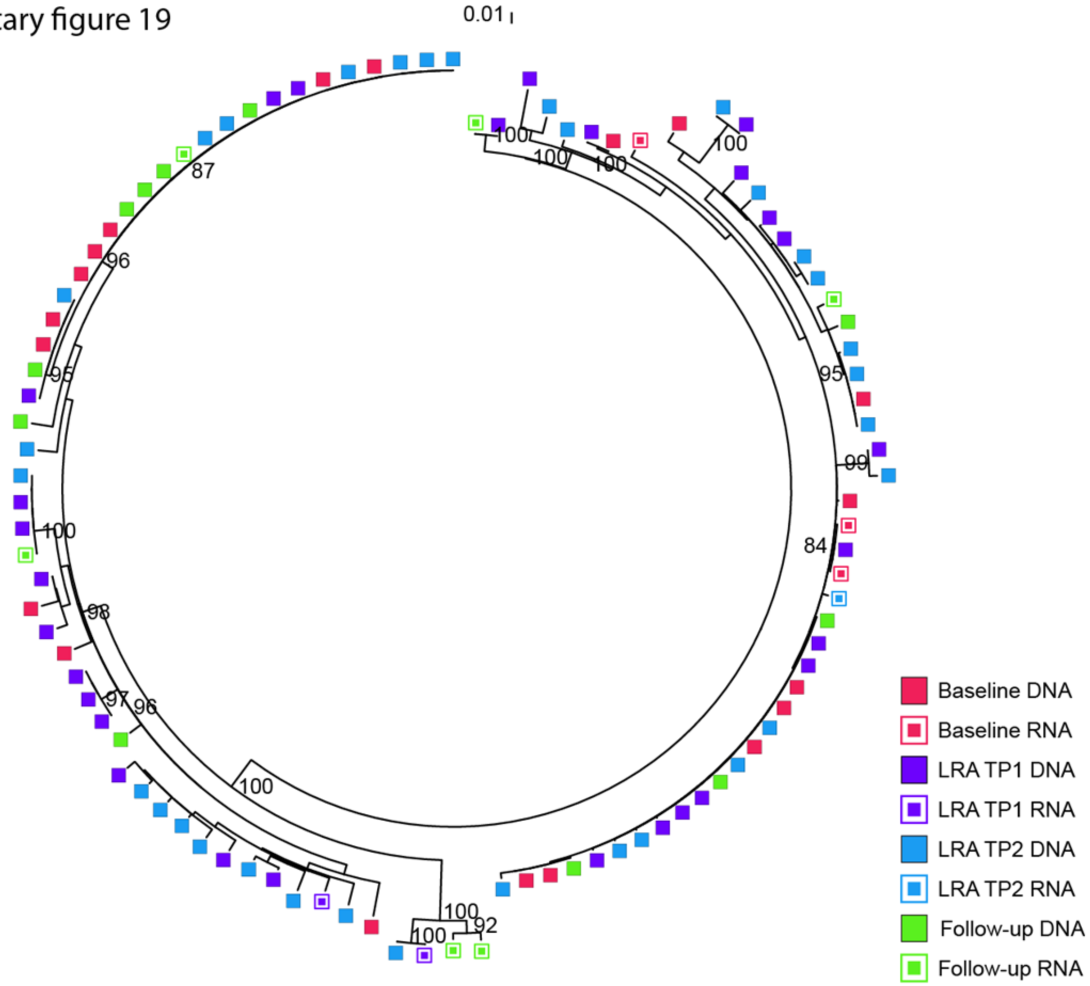

Supplementary Figure 19 Vorinostat participant VOR009 phylogenetic tree. Maximum-likelihood tree of the V1-V3 region of HIV-1 *env* sequences obtained using single-genome sequencing on isolated CD4+ T cells for participant VOR009 from the vorinostat trial. The phylogenetic analysis and tree construction were conducted using MEGA-CC. Defective sequences (hypermutants or containing stop codons) were excluded from the phylogenetic tree analysis. Peripheral blood samples were collected at baseline, 24 hours after the first dose of vorinostat (TP1), 7 days after the first dose of vorinostat (TP2), and 7 days after the final vorinostat dose.

Supplementary figure 20

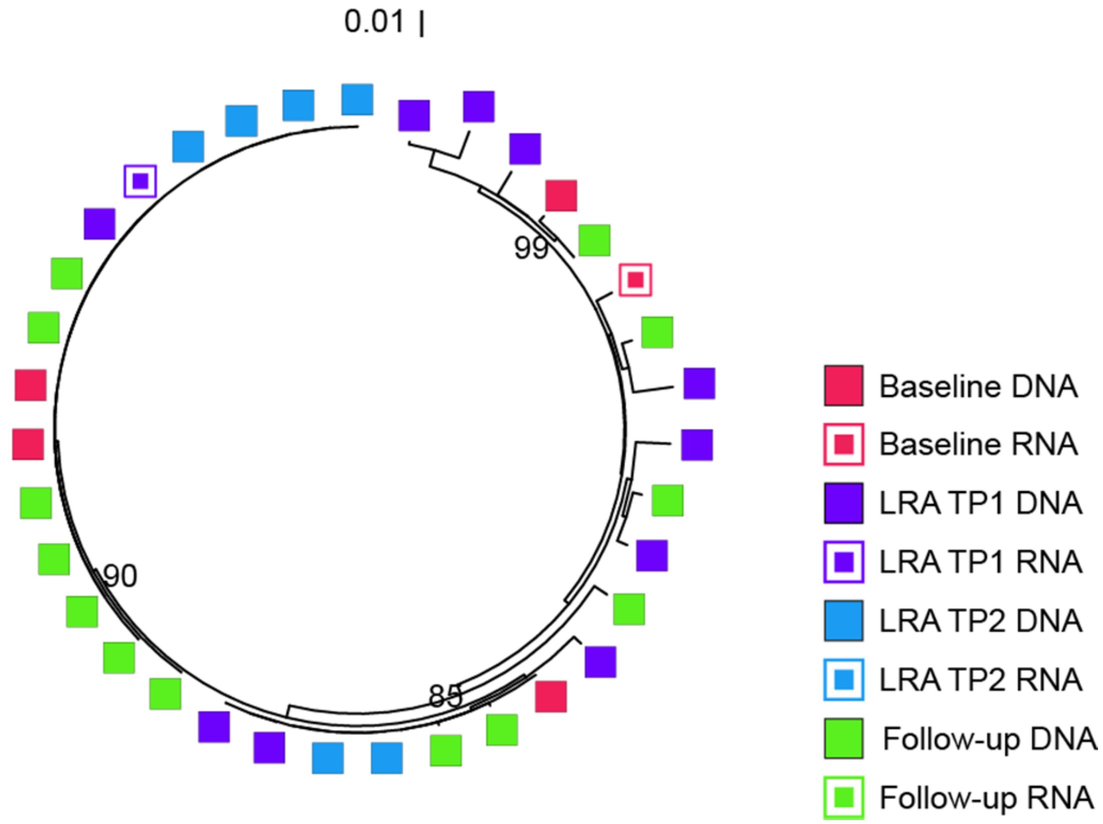

Supplementary Figure 20 Vorinostat participant VOR011 phylogenetic tree. Maximum-likelihood tree of the V1-V3 region of HIV-1 *env* sequences obtained using single-genome sequencing on isolated CD4+ T cells for participant VOR011 from the vorinostat trial. The phylogenetic analysis and tree construction were conducted using MEGA-CC. Defective sequences (hypermutants or containing stop codons) were excluded from the phylogenetic tree analysis. Peripheral blood samples were collected at baseline, 8 hours after the first dose of vorinostat (TP1), 14 days after the first dose of vorinostat (TP2), and 7 days after the final vorinostat dose.

Supplementary figure 21

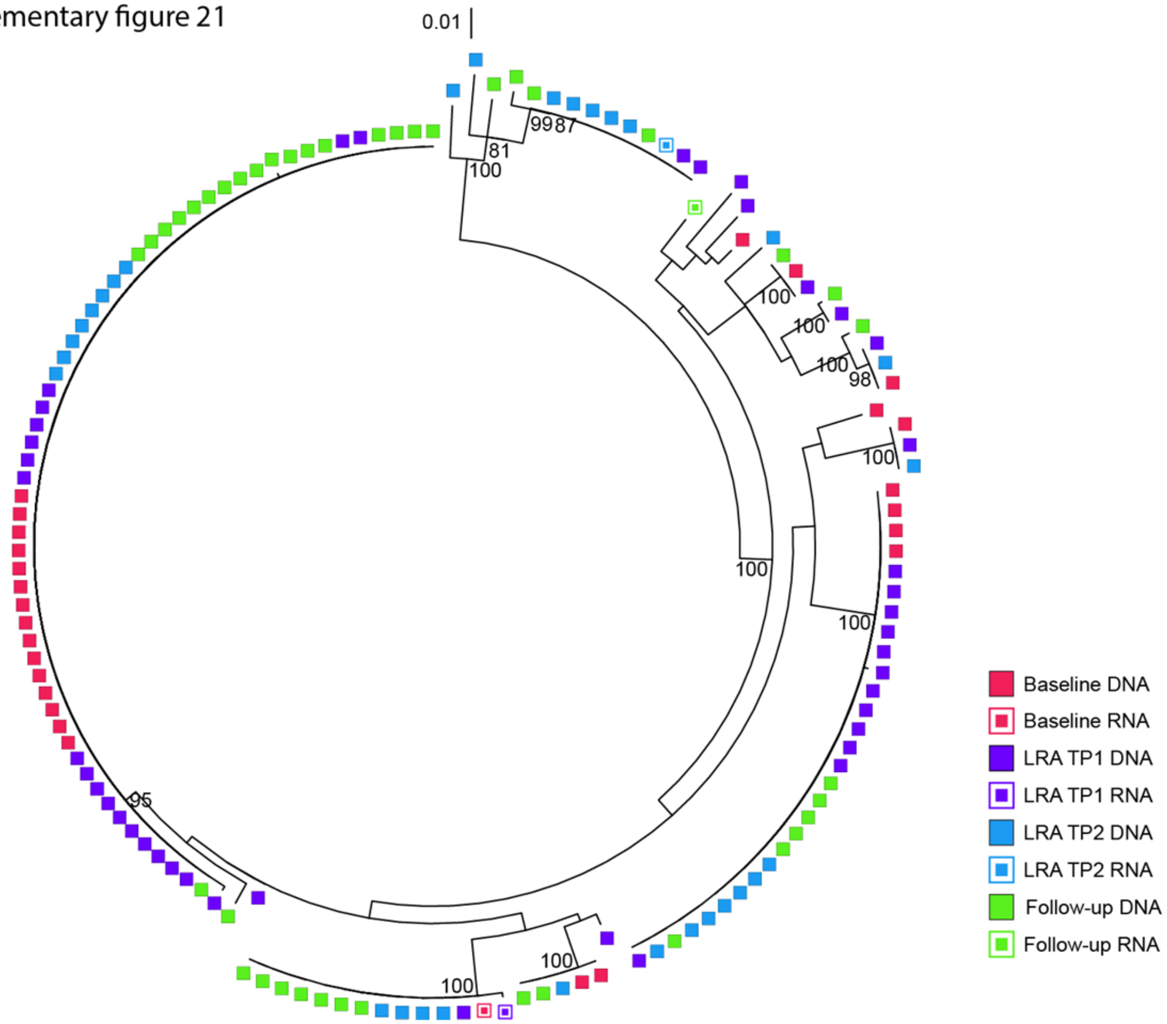

Supplementary Figure 21 Vorinostat participant VOR013 phylogenetic tree. Maximum-likelihood tree of the V1-V3 region of HIV-1 *env* sequences obtained using single-genome sequencing on isolated CD4+ T cells for participant VOR013 from the vorinostat trial. The phylogenetic analysis and tree construction were conducted using MEGA-CC. Defective sequences (hypermutants or containing stop codons) were excluded from the phylogenetic tree analysis. Peripheral blood samples were collected at baseline, 24 hours after the first dose of vorinostat (TP1), 7 days after the first dose of vorinostat (TP2), and 7 days after the final vorinostat dose.

Supplementary figure 22

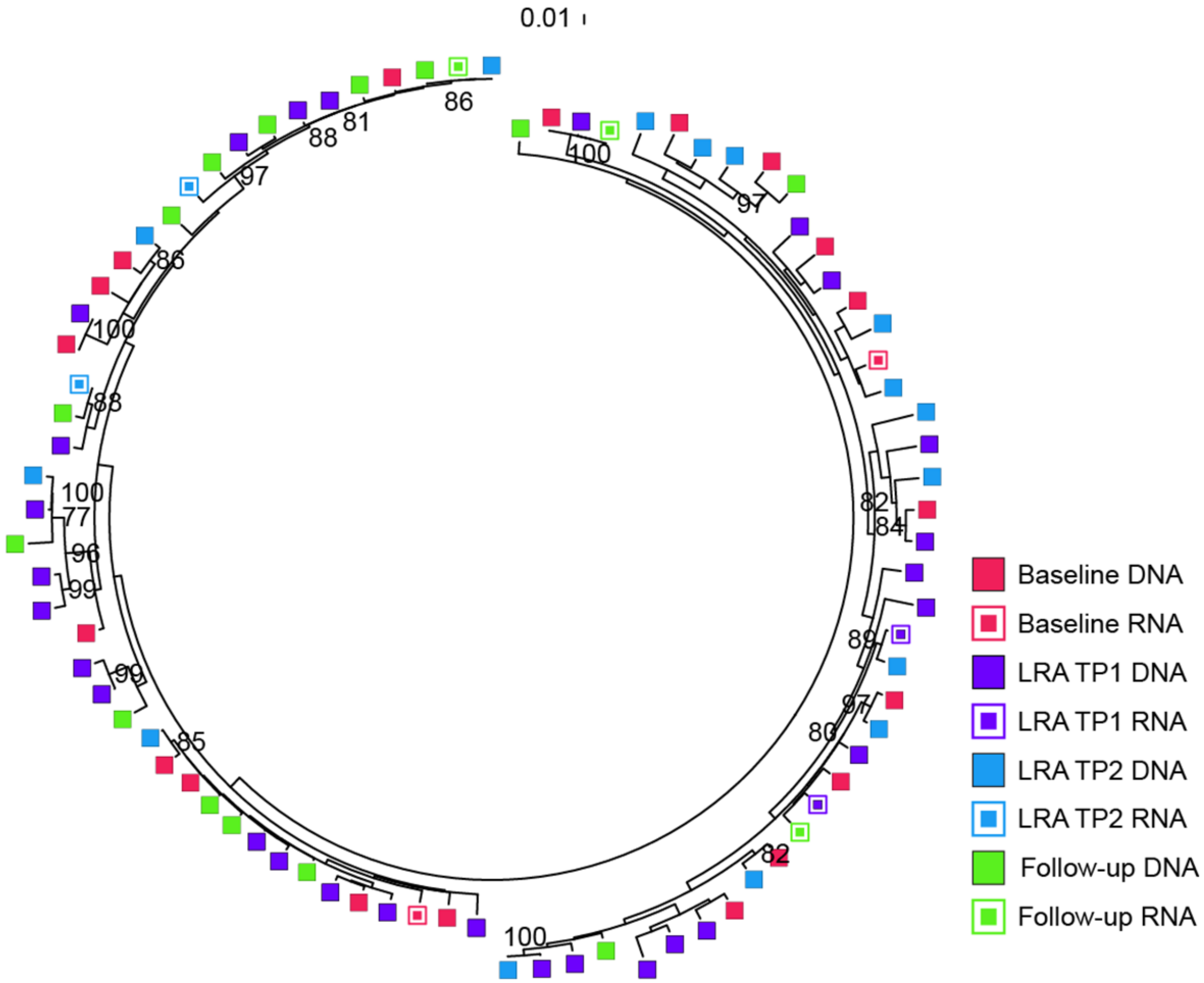

Supplementary Figure 22 Vorinostat participant VOR014 phylogenetic tree. Maximum-likelihood tree of the V1-V3 region of HIV-1 *env* sequences obtained using single-genome sequencing on isolated CD4+ T cells for participant VOR014 from the vorinostat trial. The phylogenetic analysis and tree construction were conducted using MEGA-CC. Defective sequences (hypermutants or containing stop codons) were excluded from the phylogenetic tree analysis. Peripheral blood samples were collected at baseline, 24 hours after the first dose of vorinostat (TP1), 7 days after the first dose of vorinostat (TP2), and 7 days after the final vorinostat dose.

Supplementary figure 23

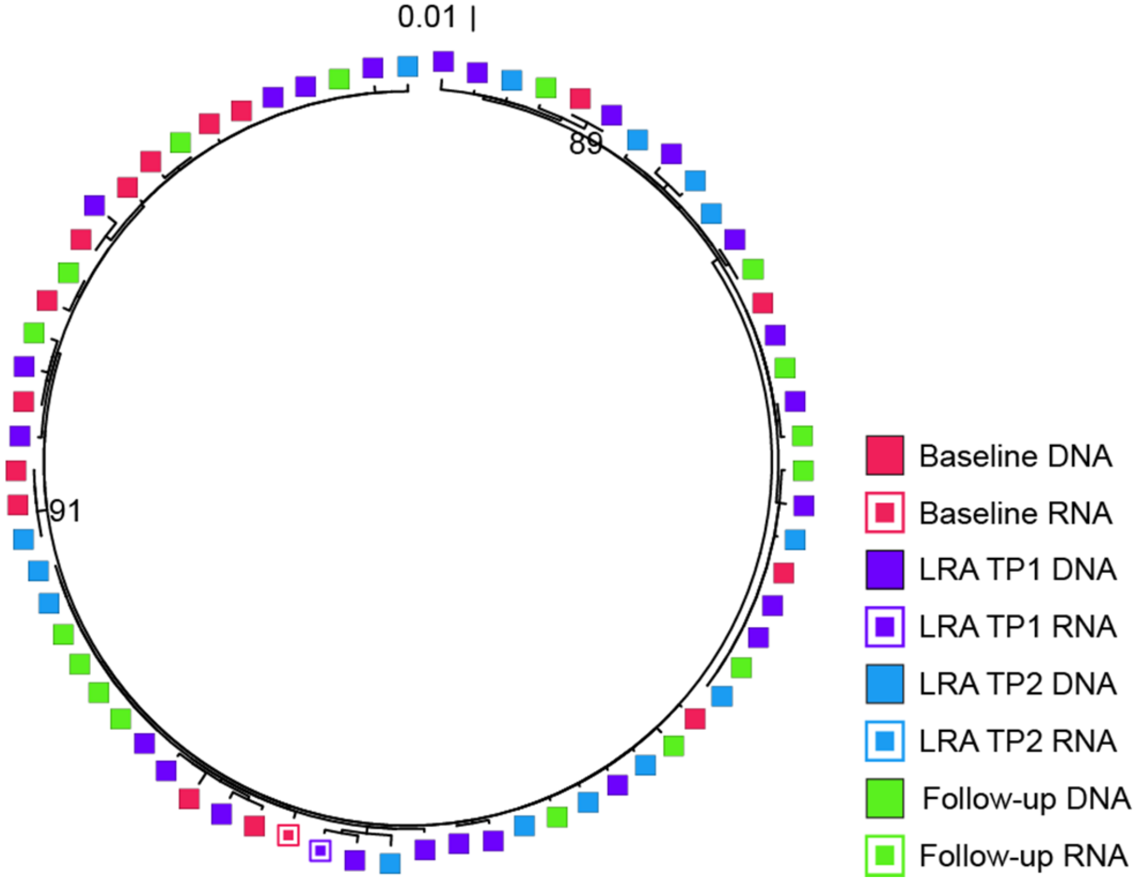

Supplementary Figure 23 Vorinostat participant VOR015 phylogenetic tree. Maximum-likelihood tree of the V1-V3 region of HIV-1 *env* sequences obtained using single-genome sequencing on isolated CD4+ T cells for participant VOR015 from the vorinostat trial. The phylogenetic analysis and tree construction were conducted using MEGA-CC. Defective sequences (hypermutants or containing stop codons) were excluded from the phylogenetic tree analysis. Peripheral blood samples were collected at baseline, 24 hours after the first dose of vorinostat (TP1), 7 days after the first dose of vorinostat (TP2), and 7 days after the final vorinostat dose.

Supplementary figure 24

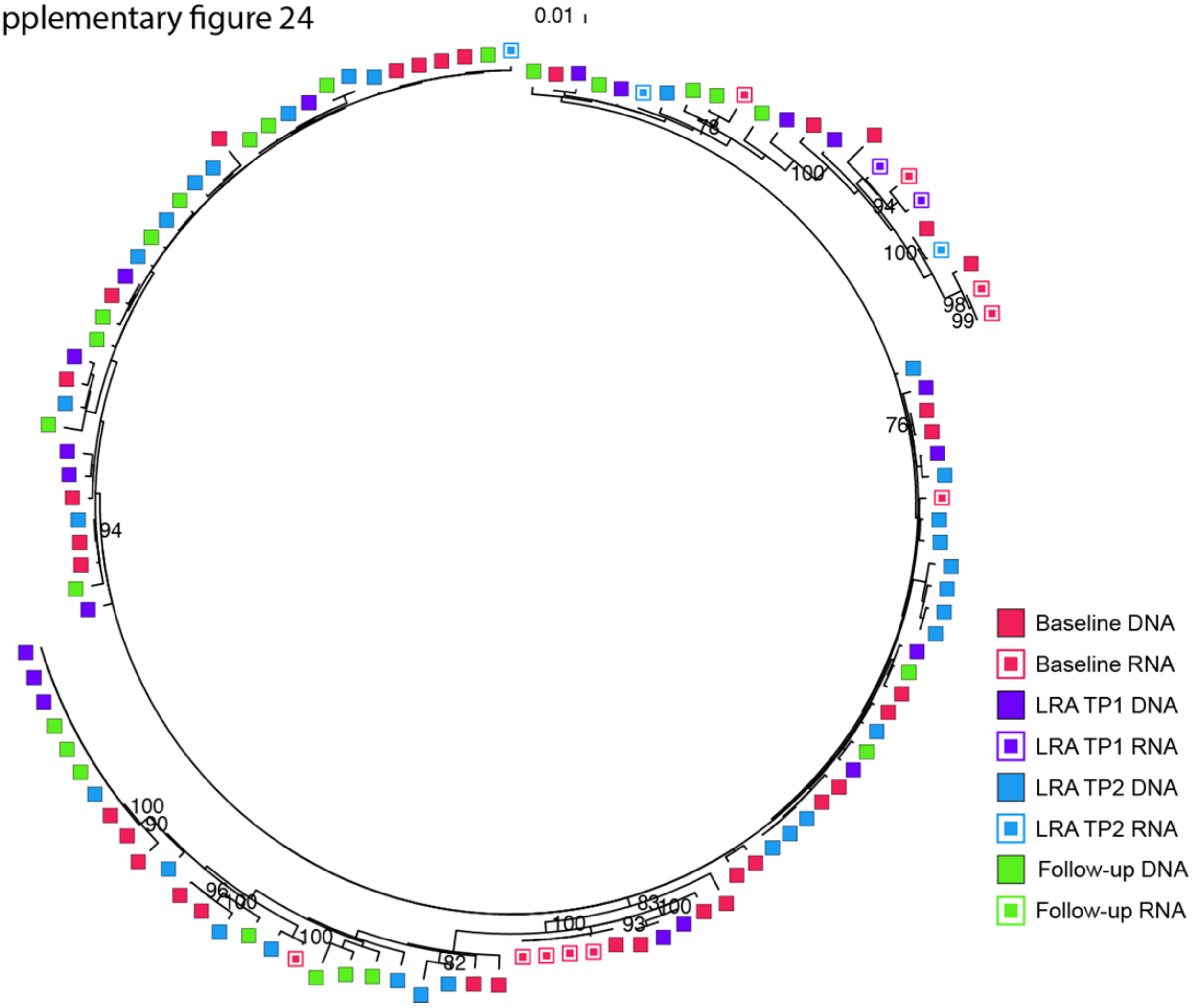

Supplementary Figure 24 Vorinostat participant VOR018 phylogenetic tree. Maximum-likelihood tree of the V1-V3 region of HIV-1 *env* sequences obtained using single-genome sequencing on isolated CD4+ T cells for participant VOR018 from the vorinostat trial. The phylogenetic analysis and tree construction were conducted using MEGA-CC. Defective sequences (hypermutants or containing stop codons) were excluded from the phylogenetic tree analysis. Peripheral blood samples were collected at baseline, 8 hours after the first dose of vorinostat (TP1), 14 days after the first dose of vorinostat (TP2), and 7 days after the final vorinostat dose.

Supplementary figure 25

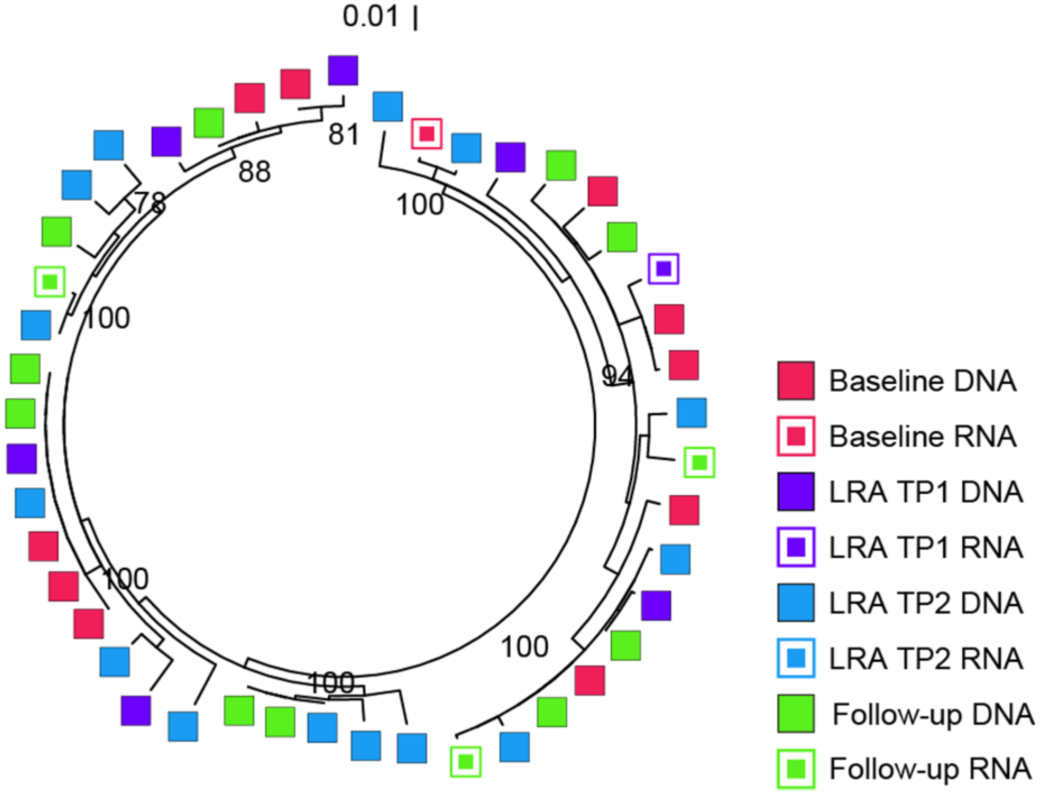

Supplementary Figure 25 Vorinostat participant VOR019 phylogenetic tree. Maximum-likelihood tree of the V1-V3 region of HIV-1 *env* sequences obtained using single-genome sequencing on isolated CD4+ T cells for participant VOR019 from the vorinostat trial. The phylogenetic analysis and tree construction were conducted using MEGA-CC. Defective sequences (hypermutants or containing stop codons) were excluded from the phylogenetic tree analysis. Peripheral blood samples were collected at baseline, 24 hours after the first dose of vorinostat (TP1), 14 days after the first dose of vorinostat (TP2), and 7 days after the final vorinostat dose.

Supplementary figure 26

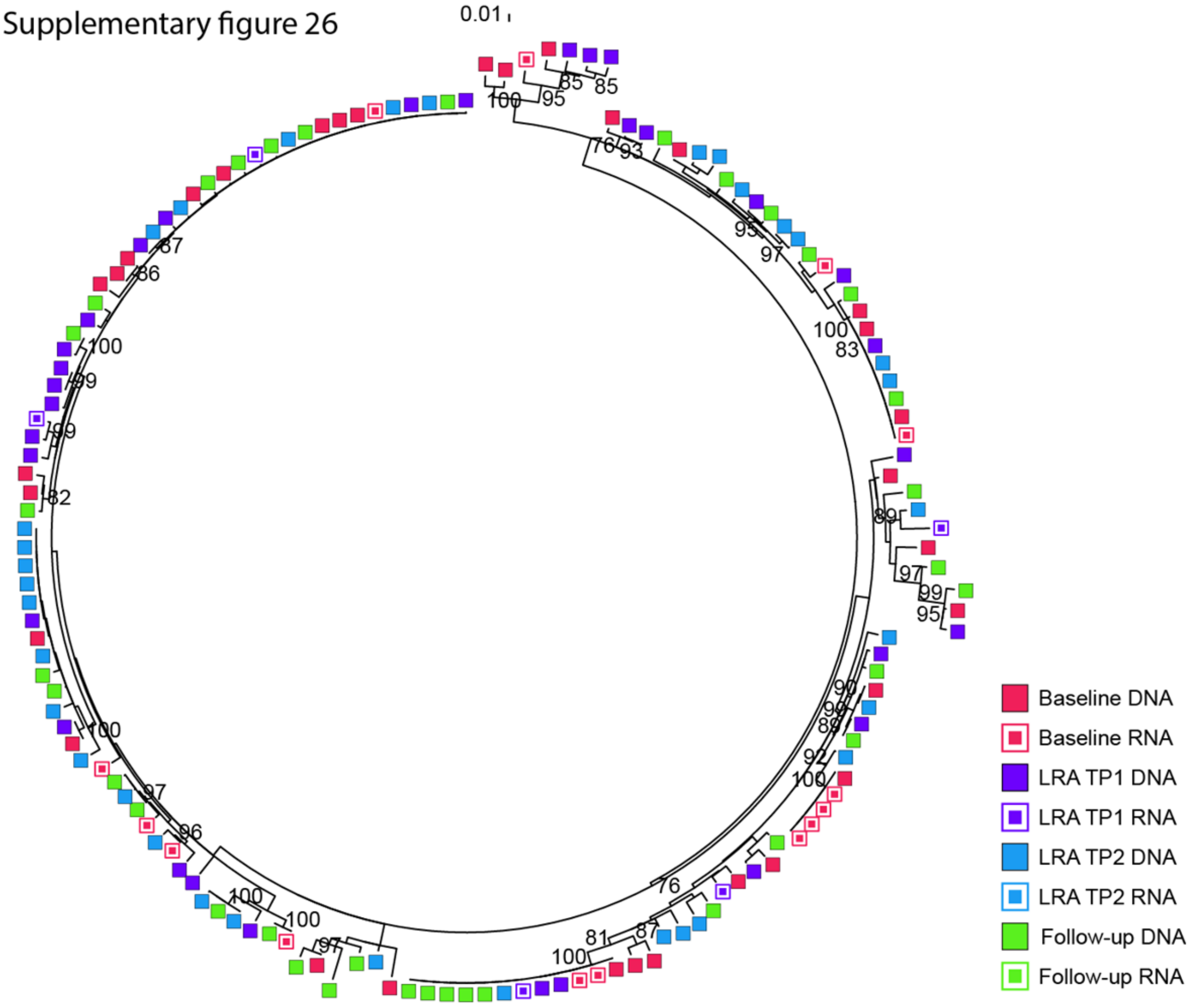

Supplementary Figure 26 Vorinostat participant VOR020 phylogenetic tree. Maximum-likelihood tree of the V1-V3 region of HIV-1 *env* sequences obtained using single-genome sequencing on isolated CD4+ T cells for participant VOR020 from the vorinostat trial. The phylogenetic analysis and tree construction were conducted using MEGA-CC. Defective sequences (hypermutants or containing stop codons) were excluded from the phylogenetic tree analysis. Peripheral blood samples were collected at baseline, 24 hours after the first dose of vorinostat (TP1), 7 days after the first dose of vorinostat (TP2), and 7 days after the final vorinostat dose.

Supplementary figure 27

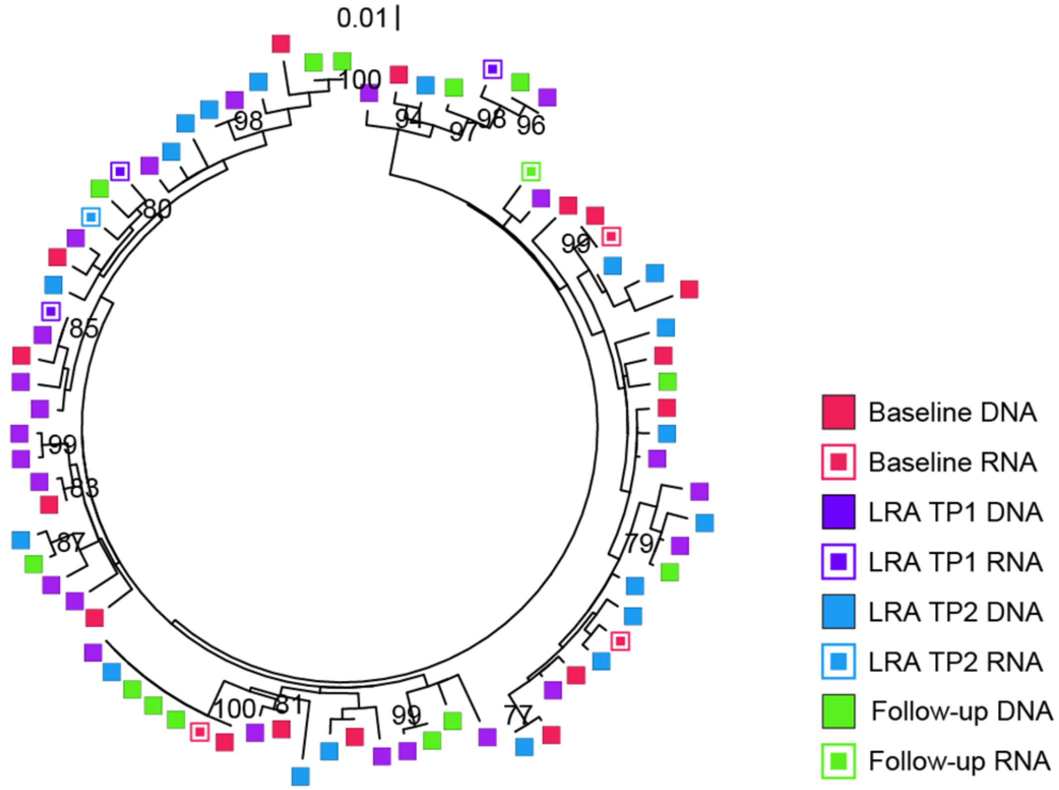

Supplementary Figure 27 Vorinostat participant VOR022 phylogenetic tree. Maximum-likelihood tree of the V1-V3 region of HIV-1 *env* sequences obtained using single-genome sequencing on isolated CD4+ T cells for participant VOR022 from the vorinostat trial. The phylogenetic analysis and tree construction were conducted using MEGA-CC. Defective sequences (hypermutants or containing stop codons) were excluded from the phylogenetic tree analysis. Peripheral blood samples were collected at baseline, 2 hours after the first dose of vorinostat (TP1), 7 days after the first dose of vorinostat (TP2), and 7 days after the final vorinostat dose.

Supplementary figure 28

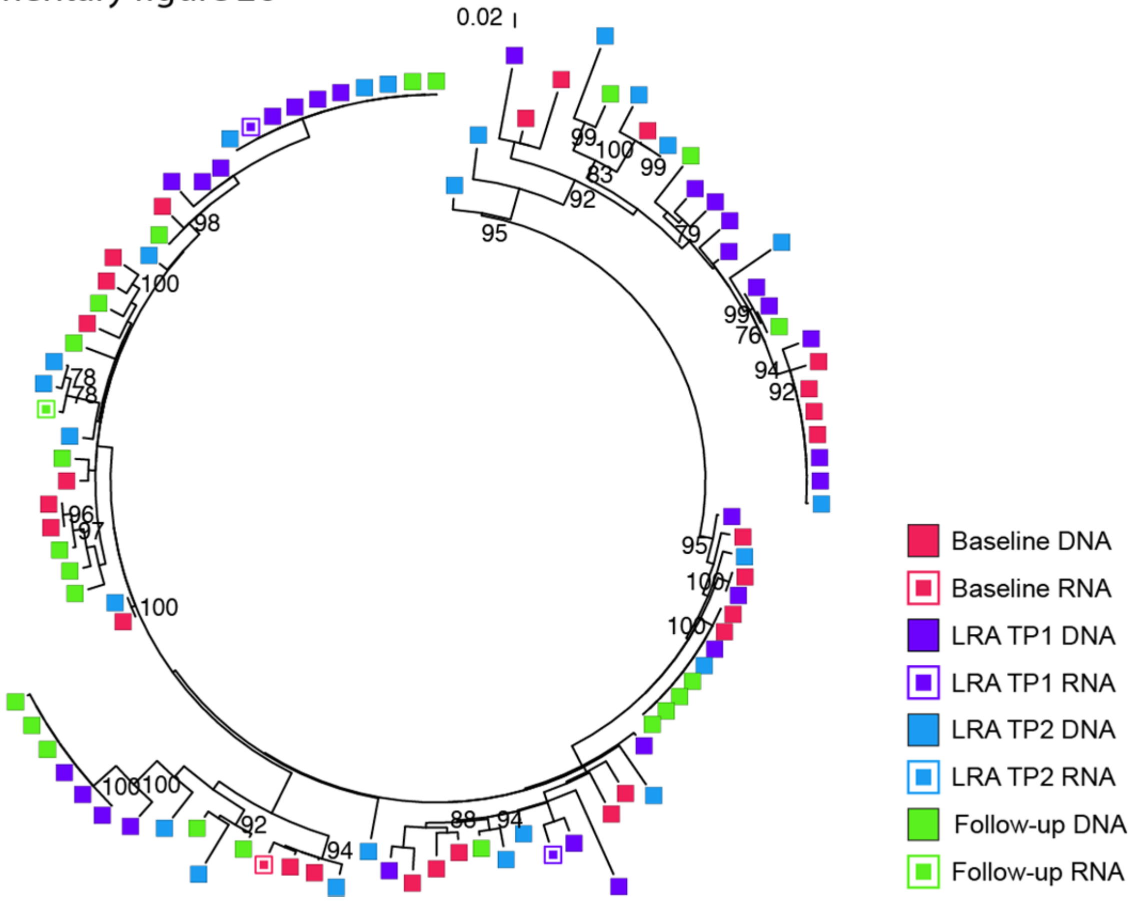

Supplementary Figure 28 Vorinostat participant VOR023 phylogenetic tree. Maximum-likelihood tree of the V1-V3 region of HIV-1 *env* sequences obtained using single-genome sequencing on isolated CD4+ T cells for participant VOR023 from the vorinostat trial. The phylogenetic analysis and tree construction were conducted using MEGA-CC. Defective sequences (hypermutants or containing stop codons) were excluded from the phylogenetic tree analysis. Peripheral blood samples were collected at baseline, 7 days after the first dose of vorinostat (TP1), 14 days after the first dose of vorinostat (TP2), and 7 days after the final vorinostat dose.

Supplementary figure 29

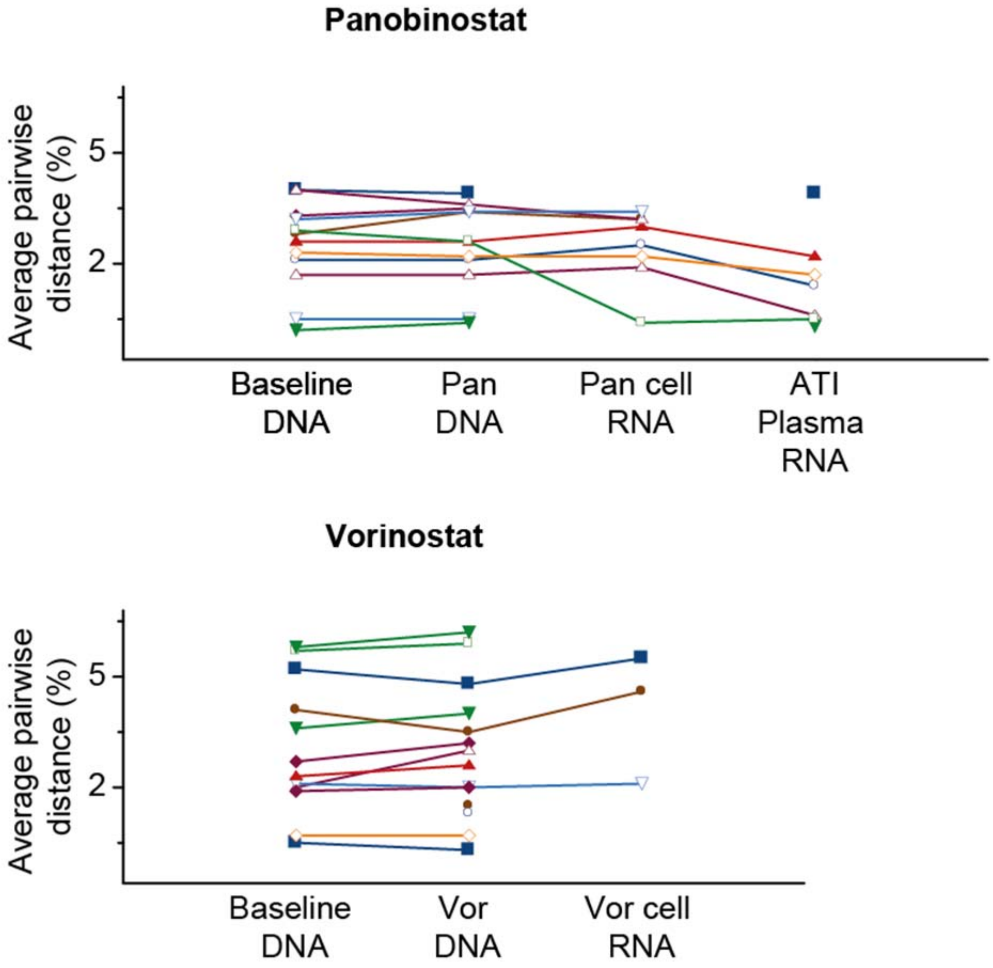

Supplementary Figure 29 Linear mixed effects model of the average pairwise distance. A linear mixed effect model was used to compare the average pairwise distance (%) of the baseline DNA, on HDACi DNA, on HDAC RNA, and analytical treatment interruption plasma RNA within each drug trial. Patient identifier was considered as a random effect and the sample type factor as both a fixed effect and as a random effect with a general positive definite covariance structure. In the Panobinostat drug trial, there was a statistically significant within patient difference in average pairwise distance (%) across the four sample types ($p<0.001$). There was no statistically significant within patient difference between the baseline and on trial DNA samples (mean change 0.02%, SE 0.08%, $p=0.741$), nor between the on trial DNA and RNA samples (mean difference 0.11%, SE 0.33%, $p=0.748$). There was however a statistically significant within patient difference between the on trial RNA measurement

and that following treatment interruption (mean change -0.72%, SE 0.55%, p=0.043). In the Vorinostat drug trial, only 3 of the 14 subjects had measurable on trial RNA values and no RNA measurements were taken following treatment interruption. There was no statistically significant within patient difference in average pairwise distance (%) across the baseline or on trial sample types (p=0.2). There was no statistically significant within patient difference between the baseline and on trial DNA samples (mean change 0.13%, SE 0.12%, p=0.298), nor between the on trial RNA and DNA samples (mean difference 0.35%, SE 0.23%, p=0.154). The linear mixed effect model was used to generate the p values.

Supplementary figure 30

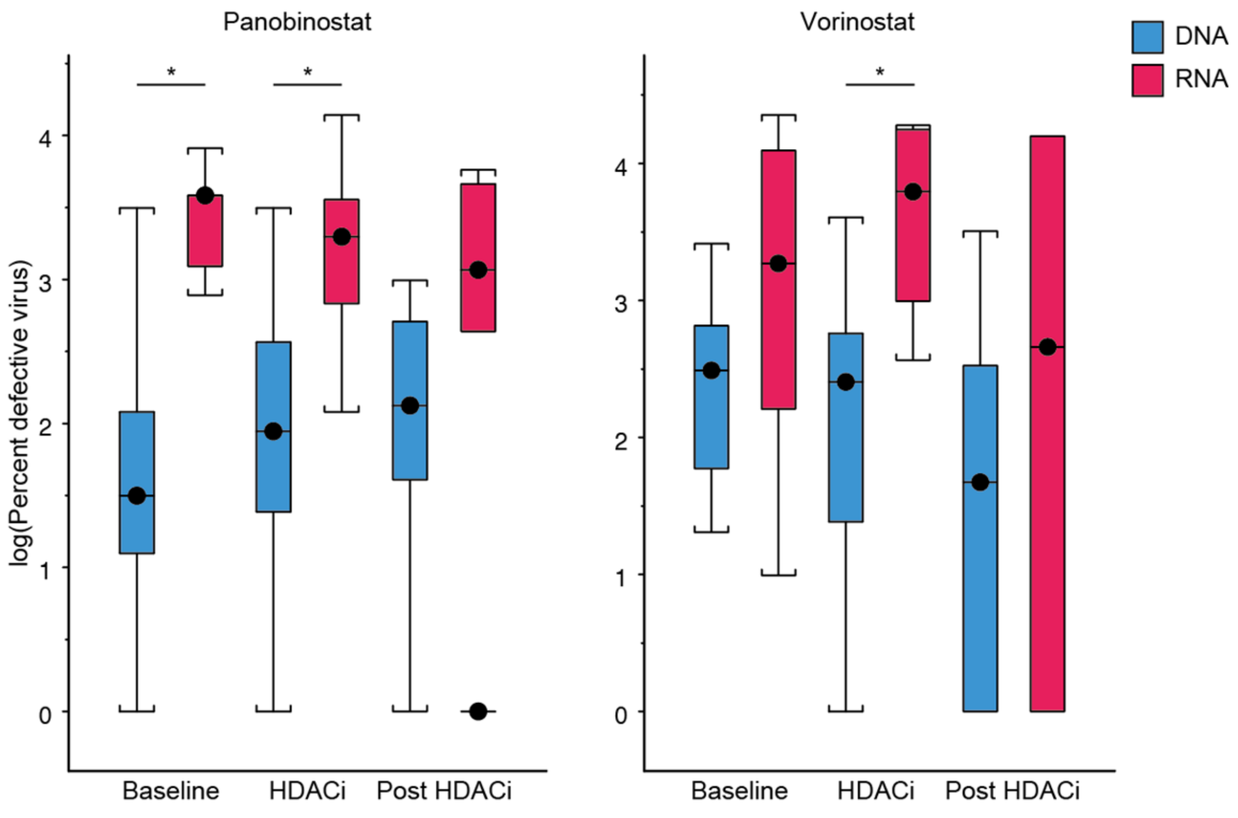

Supplementary figure 30 Linear mixed effect model of the percent defective virus. The percentage of defective virus was log transformed to approximate normality and to stabilize the variance prior to analysis. Linear mixed effects models were used to investigate the joint effects of time (treated as a 3-level factor) and type (DNA or RNA) on log(percentage of defective virus) within each drug trial. Patient identifier, time, and

type were considered as random effects with a general positive definite covariance structure, and time, type, and their 2-way interaction as fixed effects. Parameter estimates and their 95% confidence intervals (95%CI) for the log(percentage of dead-end virus) analyses were back- transformed to present results using the original scale of measurement. In the Panobinostat drug trial, there was no statistically significant interaction between the within patient effects of the time factor (baseline, on trial, followup) and type (DNA, RNA) on log(percentage defective virus) (p=0.267). The linear mixed effect model was used to generate p values.

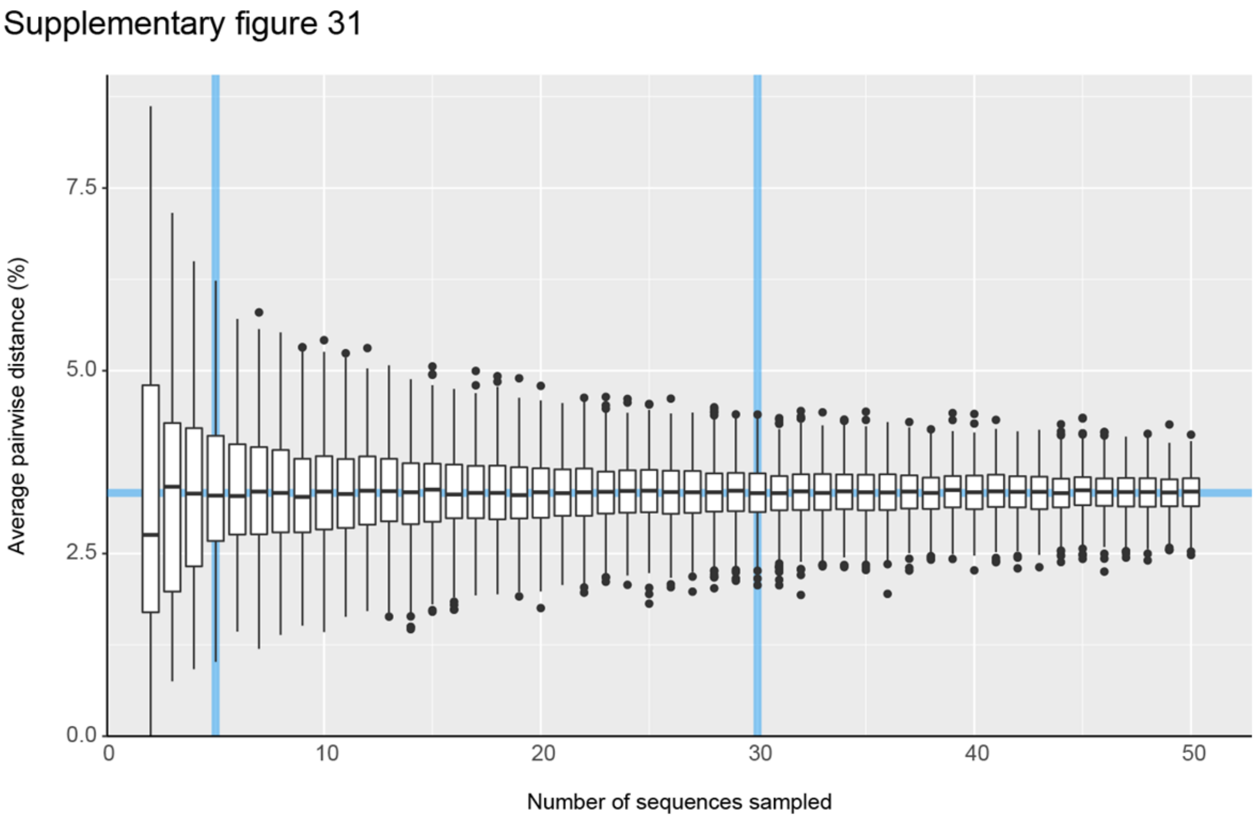

Supplementary figure 31 Simulation of sampling for the average pairwise distances. Box plot illustrating the results of a simulation of 1,000 replicate comparisons of the average pairwise distance for 2-50 sequences. Five sequences can be used to generate an accurate measure of the average pairwise distance (mean simulation 3.345 vs. actual mean 3.327, variance 1.07, 95% CI 3.281-3.410).

Supplementary table 1 Number of intact sequences obtained for each sample for each participant in the panobinostat trial.

Participant	Pre-ART plasma RNA	Baseline DNA	Baseline RNA	Time point 1 DNA	Time point 1 RNA	Time point 2 DNA	Time point 2 RNA	Follow-up DNA	Follow-up RNA	Treatment Interruption plasma RNA	Baseline LPMC DNA	Baseline LPMC RNA	Week 4 LPMC DNA	Week 4 LPMC RNA	Naïve	Central Memory	Effector Memory	Stem cell Memory	Terminally differentiated
Pan01	22	27	7	21	4	22	3	22	2	27	26	0	9	0	0	7	0	3	0
Pan02	13	36	9	49	5	69	2	25	1	29	-	-	-	-	8	21	20	16	13
Pan04	30	27	1	17	0	4	0	5	0	13	-	-	-	-	0	0	13	0	3
Pan05	-	0	0	7	0	3	0	5	0	-	13	0	2	0	-	-	-	-	-
Pan06	25	24	1	29	2	27	1	27	0	-	-	-	-	-	-	-	-	-	-
Pan07	26	27	7	28	9	27	5	24	5	-	21	0	5	0	-	-	-	-	-
Pan08	-	24	20	18	16	22	15	17	8	30	-	-	-	-	0	26	15	0	0
Pan09	-	28	1	16	0	28	1	23	1	9	6	0	1	0	24	7	28	0	0
Pan10	-	29	0	27	1	28	2	27	0	4	29	0	13	0	0	0	0	0	0
Pan12	27	5	0	8	0	9	0	4	0	26	-	-	-	-	2	1	2	0	0
Pan14	29	4	0	4	1	3	0	5	0	-	3	0	4	0	-	-	-	-	-
Pan15	26	36	3	24	11	16	6	41	6	-	16	3	13	0	0	0	0	0	0
Pan17	-	24	1	21	22	14	0	30	4	13	8	1	14	1	0	7	16	0	0
Pan18	28	28	7	27	15	29	18	28	6	24	25	14	28	8	-	-	-	-	-
Pan19	15	32	9	24	24	45	11	36	14	-	0	0	0	0	-	-	-	-	-

Supplementary table 2 Number of intact sequences obtained for each sample for each participant in the vorinostat trial as well as whether the participant began ART treatment during acute or chronic infection.

Participant	Acute/Chronic	Baseline DNA	Baseline RNA	Time point 1 DNA	Time point 1 RNA	Time point 2 DNA	Time point 2 RNA	Follow-up DNA	Follow-up RNA
Vor01	**C**	1	1	10	1	13	1	9	0
Vor02	**A**	10	0	11	0	8	0	2	0
Vor03	**C**	32	1	33	0	43	2	35	0
Vor06	**C**	22	4	13	1	16	2	8	1
Vor09	**C**	19	3	28	2	32	1	11	6
Vor11	**C**	4	1	10	1	6	0	13	0
Vor13	**C**	26	1	42	1	30	1	41	1
Vor14	**C**	19	2	28	2	15	2	14	3
Vor15	**C**	15	1	24	1	14	0	16	0
Vor16	**C**	29	36	31	13	41	7	24	8
Vor18	**C**	34	10	19	2	29	3	24	0
Vor19	**C**	10	1	6	1	14	0	10	3
Vor20	**C**	32	14	31	5	33	0	35	0
Vor22	**C**	16	3	23	3	19	1	13	1
Vor23	**C**	26	1	28	2	24	0	22	1
	**1/14**	**295**	**79**	**337**	**35**	**337**	**20**	**277**	**24**

Supplementary table 3 Linear mixed effects model of the percent defective virus for the panobinostat trial.

Time	RNA/DNA	Lower CI	Upper CI	p-value	n
**Baseline**	5.0	2.7	9.4	0.001	7
**On trial**	3.6	1.6	8.0	0.007	9
**Follow up**	2.1	0.2	19.2	0.416	6

CI-confidence interval

Supplementary table 4 Linear mixed effects model of the percent defective virus for the vorinostat trial.

Time	RNA/DNA	Lower CI	Upper CI	p-value	n
**Baseline**	1.4	0.3	6.3	0.616	5
**On trial**	3.2	1.3	8.2	0.024	6
**Follow up**	2.0	0.0	>30	0.697	3

CI-confidence interval
